# Modeling Trophic Dynamics in Lake Võrtsjärv: Energy Flow and Species Interactions

**DOI:** 10.1002/ece3.71692

**Published:** 2025-07-02

**Authors:** Maria Tirronen, Anna Kuparinen

**Affiliations:** ^1^ Department of Biological and Environmental Science University of Jyväskylä Jyväskylä Finland; ^2^ Natural Resources Institute Finland (Luke) Jyväskylä Finland

**Keywords:** allometric trophic network model, bioenergetic model, environmental noise, food web, lake ecosystem, model calibration

## Abstract

Understanding ecosystem dynamics is essential for ecological research and resource management. Bioenergetic or allometric trophic network models are effective in elucidating these interactions. However, aligning them accurately with empirical data remains challenging. Our present study contributes to such efforts by developing a trophic network model to describe population dynamics at Lake Võrtsjärv, Estonia, with a focus on predator–prey relationships and energy considerations. We calibrate this model to empirical biomass time series data using numerical optimization methods, a process previously applied to bionergetic models with considerably fewer guilds and/or parameters. Our approach emphasizes aligning the model closely with empirical time series and yields 77%–81% similarity between the modeled average dynamics and recorded biomasses. Despite relatively high similarity, the models we tested for noise—those assuming observation noise, as well as those incorporating environmental noise through stochastic differential equations—could not describe the annual variation of biomasses realistically. Overall, our tentative results demonstrate both the potential and the challenges involved in calibrating bioenergetic models to empirical data from large food webs.

## Graphical abstract

Aligning bioenergetic, or allometric trophic network, models accurately with empirical data remains challenging. Our present study contributes to these efforts by developing a trophic network model to describe population dynamics at Lake Võrtsjärv, Estonia, with a focus on predator–prey relationships and energy considerations. We calibrate this model to empirical biomass time series data using numerical optimization methods, an approach previously applied to bionergetic models with considerably fewer guilds and/or parameters.

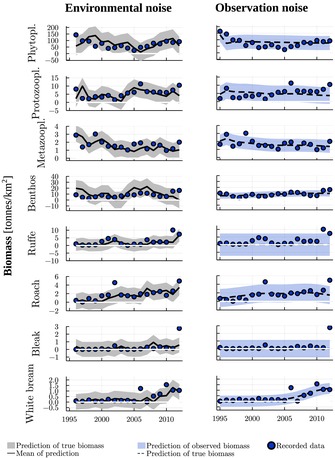

## 1 Introduction

Understanding the intricate dynamics of ecosystems is crucial for both ecological theory and practical management of natural resources. Bioenergetic, or allometric trophic network (ATN) models (Martinez 2020; Boit et al. 2012; Brose et al. 2006; Williams et al. 2007; Yodzis and Innes 1992), are at the forefront of such efforts, providing effective tools for understanding the dynamic interactions within ecosystems. These differential equation models extend the traditional Lotka‐Volterra (Lotka 1925; Volterra 1926) predator–prey model by calculating the rates of change based on metabolic and allometric rules and by adjusting predator consumption according to prey density. By incorporating the principles of energy flow and biomass distribution across various trophic levels, these models offer a general framework that enhances our understanding of species interactions (Boit et al. 2012). They not only advance basic theoretical knowledge but also provide crucial insights into the mechanisms underlying ecological stability (Brose et al. 2006) and the effects of human‐induced changes (Kuparinen et al. 2023; Perälä et al. 2023; Uusi‐Heikkilä et al. 2022).

Despite their theoretical importance, aligning ATN models accurately with empirical data remains a significant challenge. Indeed, only a few studies (Banks et al. 2017a, 2017b; Boit et al. 2012; Koen‐Alonso and Yodzis 2005) have actually tested whether modeled ATN dynamics are able to resemble empirical time series data of abundances. The first one (Koen‐Alonso and Yodzis 2005) of the studies developed a four‐species model for part of the marine community of northern and central Patagonia, Argentina, describing the dynamics of the system on an annual scale, and investigated how different model structures, such as alternative functional responses, behave under different exploitation scenarios. While the authors set allometrically derived parameters following Yodzis and Innes (1992), they utilized numerical methods to estimate other parameters and initial conditions. Overall, most of their models were able to describe reasonably well the time series for two of the four species but were less accurate for the rest.

Later, Boit et al. (2012) tested whether the ATN model can reproduce observed time series of plankton and fish species in Lake Constance during a growing season. For this, the authors extended the ATN model to include more detailed metabolism as well as detrital loop and abiotic forcing representing seasonal changes in temperature, irradiance and the level of nutrients. The model consisted of 24 functional groups (guilds). The authors calibrated prey resistance in functional responses manually and set the rest of the parameters based on literature or measurements on the site, obtaining 82% average similarity between an idealized time series of a typical growing season and their best fitting model including environmental drivers.

More recently, Banks et al. (2017a, 2017b) continued to approach ATN modeling as an inverse problem with automatic calibration. They developed an ATN model for an herbivorous pest species subjected to predation in spring barley fields, the model including temperature‐dependent growth, and estimated unknown parameters using ordinary least squares. The solution of their inverse problem converged to ecologically reasonable parameter values, however, in some cases, the authors reported challenges in obtaining a good resemblence between the predicted dynamics and the data, the model performing best in cases with relatively smooth population increase.

Our present study contributes to these efforts by developing a trophic network model to describe the population dynamics of the Lake Võrtsjärv food web (Nõges and Nõges 2012), Estonia, with a focus on predator–prey relationships and energy considerations. Specifically, our objective is to develop a model that captures annual changes in the biomasses of 14 functional guilds, incorporating detrital loop and noise. We calibrate this model to biomass time series data from Lake Võrtsjärv (Bhele et al. 2022) using numerical optimization methods, an approach previously applied to models with considerably fewer guilds and/or parameters (Banks et al. 2017a, 2017b; Koen‐Alonso and Yodzis 2005). Because of imperfect information, we do not tie the rates of change directly to allometry, like traditional bioenergetic models do. Instead, we treat the parameters as free to be estimated from the data, while using metabolic and allometric rules to constrain their range. Our approach emphasizes aligning the model closely with empirical biomass time series.

## 2 Material and Methods

We first formulated a deterministic model for the population dynamics of the Lake Võrtsjärv food web, blending aspects of both bioenergetics and ecological interactions. Second, we included noise in the model by considering environmental and observational noise separately. Next, we calibrated our model to the empirical biomass time series of Lake Võrtsjärv using numerical optimization methods. In this, we tested several choices for noise structure, food web structure, parameterization, and constraints to employ in fitting.

### 2.1 Lake Võrtsjärv Population Dynamics

Following Williams et al. (2007) and Boit et al. (2012), we modeled the energy flow through the Lake Võrtsjärv food web by a set of ordinary differential equations that describe the biomasses of producer guilds (the index set $J^{\text{prod}}$), consumer guilds $\left(J^{\text{cons}}\right)$ and detritus $\left(J^{\text{detr}}\right)$ with respect to time (t) in years:

(1) $$\frac{{\mathrm{dB}}_{i}}{\mathrm{dt}}=\overset{\text{Gain from producer growth}}{r_{i}B_{i}G_{i}\left(B_{i}\right)\left(1-s_{i}\right)}-\overset{\text{Loss}\;\mathrm{by}\;\text{consumers}}{\sum_{j\in J_{i}^{\text{cons}}}B_{j}J_{\mathrm{ji}}F_{\mathrm{ji}}\left(\bar{B}\right)},\;i\in J^{\text{prod}}$$

(2) $$\begin{array}{l} \frac{{\mathrm{dB}}_{i}}{\mathrm{dt}}=-\overset{\text{Maintenance loss}}{u_{m,i}B_{i}}+\overset{\text{Gain from resources}}{u_{a,i}B_{i}\sum_{j\in J_{i}^{\mathrm{res}}}e_{\mathrm{ij}}J_{\mathrm{ij}}F_{\mathrm{ij}}\left(\bar{B}\right)}-\overset{\text{Loss}\;\mathrm{by}\;\text{consumers}}{\sum_{j\in J_{i}^{\text{cons}}}B_{j}J_{\mathrm{ji}}F_{\mathrm{ji}}\left(\bar{B}\right)}\\ \;-\overset{\text{Catch}}{C_{i}\left(t\right)},\;i\in J^{\text{cons}} \end{array}$$

(3) $$\begin{array}{l} \frac{{\mathrm{dB}}_{i}}{\mathrm{dt}}=\overset{\text{Egested resources}\;\mathrm{by}\;\text{consumers}}{\sum_{k\in J^{\text{cons}}}B_{k}\sum_{j\in J_{k}^{\mathrm{res}}\backslash J^{\text{detr}}}\left(1-e_{\mathrm{kj}}\right)J_{\mathrm{kj}}F_{\mathrm{kj}}\left(\bar{B}\right)}+\overset{\text{Exudation}\;\mathrm{by}\;\text{producers}}{\sum_{j\in J^{\text{prod}}}r_{j}B_{j}G_{j}\left(B_{j}\right)s_{j}}\\ \;-\overset{\text{Loss}\;\mathrm{by}\;\text{detritivores}}{\sum_{j\in J_{i}^{\text{cons}}}B_{j}J_{\mathrm{ji}}F_{\mathrm{ji}}\left(\bar{B}\right)},\;i=J^{\text{detr}}. \end{array}$$

Above, $B_{i}\left(t\right)$ denotes the biomass of guild i at year t, the latter occasionally omitted, and $J_{i}^{\text{cons}}$ and $J_{i}^{\mathrm{res}}$ stands for the sets of guilds that consume or are resources of guild i, respectively.

The energy flow starts from producers, which make their own energy through photosynthesis, leading to biomass growth (the first term on the right‐hand side of (1)). Similar to Boit et al. (2012), we included exudation of the producers into our model. In (1), $r_{i}$ is the intrinsic growth rate of a producer guild i, $s_{i}$ is the fraction of exudation and $G_{i}$ is defined so that the producer guild obeys logistic growth,

(4) $$G_{i}\left(B_{i}\right)=1-\frac{B_{i}}{K}$$

with K being a system‐wide carrying capacity.

Consumers feed on producers (the second term on the right hand side of (1)), and consequently, their biomass grows (the second term on the right‐hand side of (2)). But consumers also predate each other, which yields biomass increase for the predator (again, the second term on the right‐hand side of (2)) and biomass loss for the prey (the third term on the right‐hand side of (2)). The biomass of some of the consumers decreases also because of fishing ($C_{i}$ denotes the catch of guild i in (2)). All consumers lose energy for maintenance (the first term on the right‐hand side of (2)). We included both metabolism that maintains biomass and activity respiration of producing biomass into our model following Boit et al. (2012). Specifically, in (2), $u_{m,i}$ denotes the metabolic rate (maintenance) of a consumer guild i, $u_{a,i}$ is a coefficient for activity respiration so that $1-u_{a,i}$ is respired under activity, $J_{\mathrm{ij}}$ is the maximum ingestion rate of the prey guild j by the consumer i, and $e_{\mathrm{ij}}$ is the assimilation efficiency when feeding on j. Here we assumed that all biomass lost from a resource guild gets ingested by its consumer so that the model does not include a separate coefficient for ingestion efficiency. Furthermore, in Equation ((1), (2), (3)), $F_{\mathrm{ij}}$ is the functional response for the guild i consuming the guild j defined similar to Williams (2008), but without predator interference;

(5) $$F_{\mathrm{ij}}\left(\bar{B}\right)=\frac{{\left(B_{j}/B0_{\mathrm{ij}}\right)}^{1+q_{\mathrm{ij}}}}{1+\sum_{k\in J_{i}^{\mathrm{res}}}{\left(B_{k}/B0_{\mathrm{ik}}\right)}^{1+q_{\mathrm{ik}}}},$$

where $\bar{B}$ refers to a vector of biomasses, $q_{\mathrm{ij}}$ is a functional response (FR) exponent and $B0_{\mathrm{ij}}$ is the density (biomass) of the resource guild j at which the consumer guild i achieves half its maximum feeding rate, that is, $B0_{\mathrm{ij}}$ is a half‐saturation constant.

Similar to Boit et al. (2012), we included detrital loop (3) in our model, which represents dead particulate derived from producer exudation (the second term on the right‐hand side of (3)) and excretion and egestion by the consumers (the second term on the right‐hand side of (3)). Consumers may also feed on detritus (the last term in (3)).

Our model ((1), (2), (3)) describes trophic dynamics in Lake Võrtsjärv considering species interactions and the energy transfer between different levels of the food web. Unlike traditional bioenergetic models, we did not tie the producer growth rates ($r_{i}$), consumer metabolic rates ($u_{m,i}$), or ingestion rates ($J_{i,j}$), directly to species' body mass because of imperfect information (Section 2.5). We nonetheless used allometry to derive lower bounds for the metabolic rates (Section S1).

### 2.2 Environmental Noise

Previous ATN studies that modeled population dynamics during a growth season included deterministic abiotic drivers (Banks et al. 2017a, 2017b; Boit et al. 2012). We modeled the Lake Võrtsjärv dynamics on an annual scale and employed a stochastic process to describe the unpredictable combined effect of abiotic drivers on the Lake Võrtsjärv population dynamics. That is, we considered the system under environmental noise.

For the Lake Võrtsjärv population dynamics with environmental noise, we considered two stochastic differential equation (SDE; Särkkä and Solin (2019); Panik (2017)) models similar to ones suggested for the Lotka‐Volterra model (Arató 2003). Specifically, we described environmental noise in the system by a vector of independent Wiener processes. The Wiener process (or Brownian motion) is an abstraction of a random walk process that has the property that each increment is independent (Särkkä and Solin 2019). It is a real‐valued continuous‐time stochastic process that has found many applications.

In the first case, we introduced stochasticity to the dynamics by defining noise relative (proportional) to the current biomass. For each guild, we formulated the SDE describing the noisy dynamics as

(6) $${\mathrm{dB}}_{i}=f_{i}\left(\bar{B}\right)\mathrm{dt}+\sigma_{i}B_{i}{\mathrm{dW}}_{i}$$

where the drift function (Särkkä and Solin 2019) $f_{i}$ equals to the right hand side of the deterministic model of guild i dynamics in equation (1), (2), (3), $\sigma_{i}$ is a guild‐specific variance parameter and $W_{i}$ is the Wiener process. The drift function determines the nominal dynamics of the system, while the second term in (6) represents noise entering the system. In the second case, we described the noisy dynamics using absolute noise,

(7) $${\mathrm{dB}}_{i}=f_{i}\left(\bar{B}\right)\mathrm{dt}+\sigma_{i}{\mathrm{dW}}_{i}$$

the model otherwise similar to Equation 6.

Our aim was to infer the parameters of the stochastic population dynamics models (6) and (7) as an inverse problem from recorded biomasses. For this, $B_{i}^{*}\left(t\right)$ denotes the recorded value of the biomass of guild i at year t. Overall, our time series data consisted of vectors of recorded biomasses in time; ${\bar{B}}^{*}\left(t_{k}\right)$, k=1,…,T. Due to the Markov properties of SDEs, the likelihood of the observed biomasses given the parameters can be written down as (Särkkä and Solin 2019)

(8) $$L\left[{\bar{B}}^{*}\left(t_{1}\right),\ldots ,{\bar{B}}^{*}\left(t_{T}\right)|\bar{\theta }\right]=\prod_{k=1}^{T-1}\;p\left[\left.{\bar{B}}^{*}\left(t_{k}+1\right)\right|{\bar{B}}^{*}\left(t_{k}\right),\bar{\theta }\right]$$

where $p\left[{\bar{B}}^{*}\left(t_{k}+1\right)\left|{\bar{B}}^{*}\left(t_{k}\right),\bar{\theta }\right.\right]$ is the transition density of the SDE (Särkkä and Solin 2019). The model parameters are denoted by $\bar{\theta }$ for simplicity.

The evaluation of the transition density in (8) is intractable, and we proceeded by replacing the transition density with approximations. We took a common approach and discretized our model using the Euler‐Maruyama scheme, which yields an evaluable transition density (Särkkä and Solin 2019). For the model with relative noise 6, the Euler‐Maruyama scheme leads to the approximation (Särkkä and Solin 2019)

(9) $$p\left[{\bar{B}}^{*}\left(t_{k}+1\right)\left|{\bar{B}}^{*}\left(t_{k}\right),\bar{\theta }\right.\right]=\prod_{i}N\left[B_{i}^{*}\left(t_{k}+1\right)\left|B_{i}^{*}\left(t_{k}\right)+f_{i}({\bar{B}}^{*}\left(t_{k}\right),\bar{\theta })\Delta t;\sigma_{i}B_{i}^{*}\left(t_{k}\right)\sqrt{\Delta t}\right.\right]$$

where $N\left[x|y;z\right]$ denotes the Gaussian density with mean y and standard deviation z evaluated at x. The time step in our model is 1 year; Δt=1. The statistical properties of the transition density follow from the properties of the Wiener process (Särkkä and Solin 2019). For the model with absolute noise 7, the transition density is similar (Equation S1). In the following, these models are referred to as relative and absolute normal environmental noise models.

The discretized model with environmental noise predicts biomasses from the previous recorded values of biomass (equation (8)). The prediction may assign a considerable probability to biomass values ≤0. A biomass value ≤0 would clearly mean extinction of the population concerned. However, such predictions did not affect the simulation, as we always predicted from recorded biomass values that were >0. As such, we did not remove the guild from our model, even if a predicted biomass value was ≤0, but kept the food web structure the same through the whole simulation period.

In addition to the SDE models (6) and (7) driven by the Wiener process, we considered statistical models, in which the Gaussian transition density in (9) is replaced by a lognormal density, which keeps all the biomasses strictly positive. Similar to ((6), (7)), we considered relative and absolute lognormal environmental noise.

We considered only environmental noise as process noise in our model. While environmental noise is present in natural populations of all sizes, demographic noise tends to average out in large populations (Boettiger 2018; Panik 2017; Lande et al. 2003; May 1974). We regarded the Võrtsjärv guilds as large populations.

### 2.3 Observation Noise

We also considered a model in which there is no stochasticity in the process itself but the biomasses are observed with error, following previous approaches (Banks et al. 2017a, 2017b; Koen‐Alonso and Yodzis 2005). Similar to our population dynamics model with environmental noise, we approximated the solution of ((1), (2), (3)) using the Euler method (Särkkä and Solin 2019):

(10) $$B_{i}\left(t_{k}+1\right)=B_{i}\left(t_{k}\right)+f_{i}\left(\bar{B}\left(t_{k}\right),\bar{\theta }\right)\Delta t$$

starting from an initial value $\bar{B}\left(t_{1}\right)$. This may produce a biomass value ≤0, and in such a case, to avoid irregularities in our models, we did not proceed to predict the next biomass value from a non‐positive one but removed the guild from the first year of occurrence onwards.

We considered relative and absolute Gaussian noise as models of observation noise. The likelihood of the data reads as

(11) $$L\left[{\bar{B}}^{*}\left(t_{1}\right),\ldots ,{\bar{B}}^{*}\left(t_{T}\right)|\bar{\theta }\right]=\prod_{k=1}^{T}\prod_{i}N\left[B_{i}^{*}\left(t_{k}\right)|B_{i}\left(t_{k}\right),\sigma_{i}B_{i}\left(t_{k}\right)\right]$$

for relative normal noise and is similar for absolute normal noise (Equation S2).

### 2.4 Lake Võrtsjärv Data

We calibrated our models to the biomass time series of Lake Võrtsjärv (Bhele et al. 2022). The original data consisted of biomass estimates (tonnes/km^2^) of 15 guilds and detritus during 1983–2019. Bhele et al. (2022) provide full description of the data. The guilds included two phytoplankton guilds (small and large), protozooplankton, metazooplankton and benthos as well as 10 fish guilds: ruffe (*Gymnocephalus cernua*), roach ( *Rutilus rutilus* ), bleak ( *Alburnus alburnus* ), white bream ( *Blicca bjoerkna* ), bream ( *Abramis brama* ), smelt ( *Osmerus eperlanus* ), perch ( *Perca fluviatilis* ), eel ( *Anguilla Anguilla* ), pikeperch ( *Sander lucioperca* ) and pike ( *Esox lucius* ). Although phytoplankton was divided into two guilds in the original data, we included a single phytoplankton guild in the model by combining the two original phytoplankton guilds so that the plankton guilds in our model corresponded to the diet composition matrix by Cremona et al. (2018). For five of the fish guilds, the amounts of catch (tonnes/km 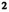) were also available during the period.

**FIGURE 1:**
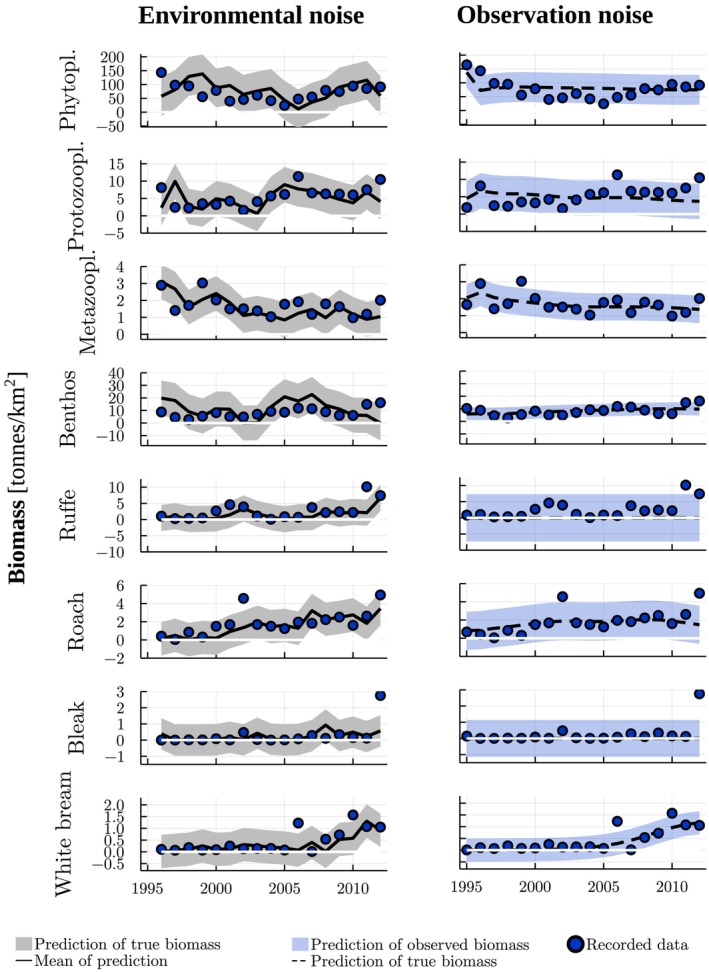
Recorded (Bhele et al. 2022) and interpolated biomass (tonnes/km^2^) data used in model fitting, and the predicted biomasses for different guilds (Table S1) by the Lake Võrtsjärv trophic model with absolute normal environmental (figures on the left) and observation noise (figures on the right). For environmental and observation noise, the feeding matrix includes and excludes, respectively, cannibalism of perch and pike and white bream as a resource for eel. The functional response exponents were set to 0.3. Both models were fitted using a penalty for biomass values ≤0. The filled areas correspond to the 90% central probability intervals.

**FIGURE 2:**
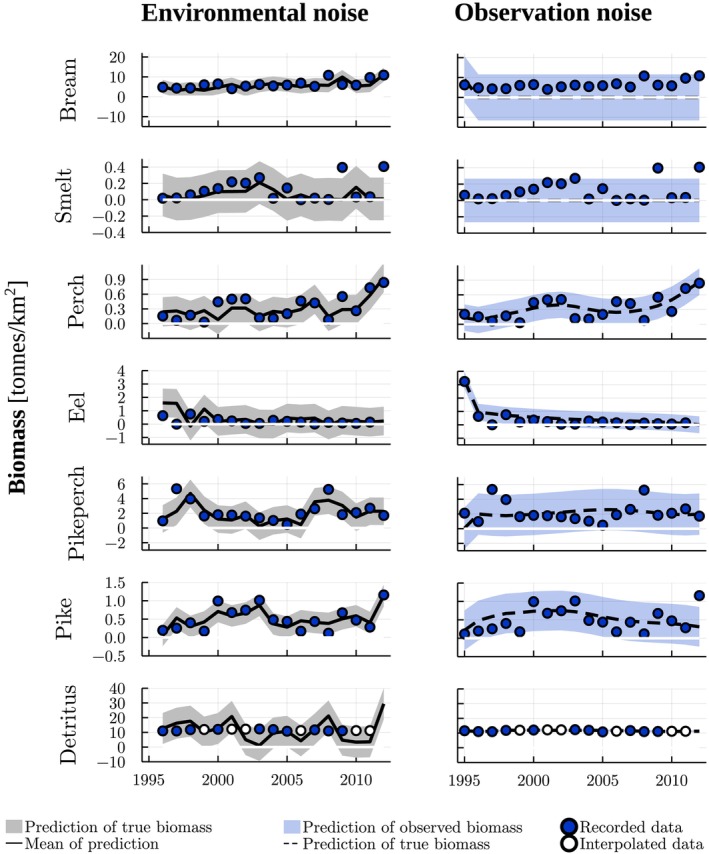
Recorded (Bhele et al. 2022) and interpolated biomass (tonnes/km^2^) data used in model fitting, and the predicted biomasses for different guilds (Table S1) by the Lake Võrtsjärv trophic model with absolute normal environmental (figures on the left) and observation noise (figures on the right). For environmental and observation noise, the feeding matrix includes and excludes, respectively, cannibalism of perch and pike and white bream as a resource for eel. The functional response exponents were set to 0.3. Both models were fitted using a penalty for biomass values ≤0. The filled areas correspond to the 90% central probability intervals.

There were several missing values in the biomass time series, particularly for protozooplankton, eel and detritus. For model fitting, we extracted only the data between 1995 and 2012. The years 1995–2011 were the longest period for which the biomasses of both protozooplankton and eel were available. The biomass estimate of protozooplankton was available also for 2012. For eel and detritus, the year 2012 was missing and thus, we ignored the predictions of eel and detritus biomasses for the year 2012 in the likelihood. In addition, this period lacked some other years for detritus but, since the biomass of detritus remained relatively constant in the system during the available years, we approximated these other missing values by the average of the nearest years before and after the missing ones. There were also two records of zero biomass (in 2006 for smelt and 1997 for eel). We interpreted these zeroes as indicators of low biomass and, to avoid irregularities in our models, replaced them with a small positive value ($10^{-8}$). Overall, we had 268 biomass estimates in our data set, six of them interpolated for detritus (Figures 1, 2).

### 2.5 Parameter Estimation

We tried to align our model closely with the Lake Võrtsjärv biomass time series by estimating model parameters as an inverse problem. In this, we tested different food web structures (Table S1), parameterizations, and constraints for fitting, in addition to different noise structures (Sections 2.2, 2.3). In regard to parameterization, we tested our model ((1), (2), (3)) with and without activity respiration.

We considered two kinds of inverse problems for the Lake Võrtsjärv model: (1) we estimated all parameters except the fraction of exudation ($s_{i}$), the assimilation efficiencies ($e_{\mathrm{ij}}$), and the acitivity respiration ($u_{a,i}$), when included in the model, and (2) we set the functional response exponents, $q_{\mathrm{ij}}$, as fixed parameters along with $s_{i}$, $e_{\mathrm{ij}}$, and $u_{a,i}$. Because there was the detrital loop, one producer and 13 other guilds, and the feeding matrix consisted of 72–75 links for the Lake Võrtsjärv food web (Table S1), the number of estimated parameters was 246–249 in case (1) and 174–177 in case (2) for our model with environmental noise. For the model with observation noise, we also needed to estimate the initial biomasses so that the respective numbers were 261–264 and 189–192.

For $s_{i}$, $e_{\mathrm{ij}}$ and $u_{a,i}$, we set values from previous studies (Boit et al. 2012; Williams et al. 2007, Table S2). To estimate the rest of the parameters, we set initial values for the carrying capacity of producers (K), half‐saturation constants ($B0_{\mathrm{ij}}$), the variance parameters ($\sigma_{i}$) and the initial biomasses ($B_{i}\left(0\right)$) based on the data (Table S3). For $q_{\mathrm{ij}}$ we set, in case (1), an initial value that was in a range likely to produce stable dynamics (Williams and Martinez 2004). In case (2), we tested different values for $q_{\mathrm{ij}}$ (Table 1). For other estimated parameters, we set initial values to one.

**TABLE 1:** Results for different noise structures and functional response exponents ($q_{\mathrm{ij}}$).

Noise	$q_{\mathrm{ij}}$	Δ AIC	Predictions ≤0	BC	90% CPI cov.
Abs. norm. env.	Estimated	11.96	17 (11)	21% %	91%
Abs. norm. env.	0	34.44	32 (31)	22%	92%
Abs. norm. env.	0.3	0	16 (15)	21%	91%
Abs. norm. env.	0.3	*42.94	*2 (2)	*23%	*92%
Abs. norm. env.	0.5	12.07	24 (24)	33%	90%
Abs. norm. env.	0.7	−19.63	23 (22)	23%	90%
Abs. norm. env.	1	−9.09	23 (23)	26%	89%
Abs. lognorm. env.	0.3	−70.93	—	26%	89%
Rel. norm. env.	Estimated	3.37 · 10^7^	190 (187)	69%	98%
Rel norm. env.	0.3	8.56 · 10^15^	127 (116)	32%	16%
Abs. norm. obs.	0.3	*18.46	*17 (17)	*19%	*92%
Rel. norm. obs.	0.3	*−255.65	*83 (83)	*19%	*37%

*Note:* Δ AIC denotes the difference in AIC value to the one obtained with absolute normal environmental noise and the functional response exponents set to 0.3. Predictions ≤0 consider the median biomass values (excluding detritus and for all guilds, the first one in parenthesis). The models with the smallest number of median biomass values ≤0 are highlighted. BC denotes the Bray–Curtis dissimilarity. 90% CPI coverage refers to the percentage of guild‐specific annual predictions, the 90% central probability interval of which covered the data. For environmental and observation noise, the feeding matrix (Table S1) includes and excludes, respectively, cannibalism of perch and pike and white bream as a resoure for eel. The model includes no activity respiration and the lower bounds of the metabolic rates of zooplanktons and benthos were set to one. The results obtained by using penalization for biomass values ≤0 are marked by *. We tested lognormal noise by continuing the search from the parameter values obtained for absolute normal environmental noise with $q_{\mathrm{ij}}=0.3$ by truncating the remaining negative biomass values to $10^{-8}$.

We carried out parameter estimation in ${\mathbb{R}}^{n}$ and transformed the estimated parameters by $\log\left(1+\exp\left(x\right)\right)$ to be strictly positive. We did not bound any parameter values from above but we derived strictly positive lower bounds, $u_{m,i}^{L}$, for the metabolic rates based on previous studies (Williams 2007; Darwall et al. 2010) and parameterized them as $u_{m,i}^{L}+u_{m,i}$. We calculated the lower bounds for the fish guilds using the species' maximum published weights (Froese and Pauly 2023) and allometric rules following Williams et al. (2007) (Table S1 and Equation S3). Because of the uncertainty about the composition of zooplankton and benthos guilds, we did not try to estimate such lower bounds for these guilds using body masses but set them to one. We also tested a higher value of the lower boundary for these guilds (Tables S6 and S12).

We carried out maximum likelihood estimation. In this, we sought for parameter values that minimize the negative log‐likelihood of the data. To avoid solutions which predict values ≤0 for the biomasses, regarded as unrealistic, we also tested loss functions that penalize for such values. The aim of these penalties was to push the model away from assigning non‐positive values to predicted biomasses. Specifically, we added the term $100n_{\leq }$ to the loss function, where $n_{\leq }$ is the number of guild‐specific annual predictions for which a criterion for non‐positivity (Table S8) holds. We chose the coefficient so that the penalization term was on the same scale as the original loss function when $n_{\leq }$ was small. We also tested a hard penalty by setting the value of the loss function to infinity if any predicted biomass was non‐positive, but, by doing so, we did not find a solution in the search space as the loss function remained infinite.

To minimize the loss functions, we used an evolutionary optimization method called the Covariance Matrix Adaptation Evolution Strategy (CMA‐ES; Hansen 2016). We implemented the models in the programming language Julia and used the package *Evolutionary.jl* (Wilde et al. 2021) for CMA‐ES. In optimization, we used 200 offspring. Although evolutionary optimization methods use a set of candidate values in the search, we only considered the best candidate of the population of solutions for simplicity. Moreover, we set the maximum number of iterations in each run of CMA‐ES to 3000, and restarted the search several times from the previously obtained optimal parameter values. For all of the models, we first run CMA‐ES with 1000 restarts. For the most potential models based on these initial results, we extended the parameter estimation by carrying out 3000 restarts in total. Except the number of offspring and iterations, we used the default parameter values for CMA‐ES in *Evolutionary.jl*.

### 2.6 Model Comparison and Similarity With Data

We compared models using the Akaike information criterion (AIC, Equation S4). According to AIC, the preferred model is the one with the lowest AIC value. Nonetheless, we also compared the models based on the number of predicted annual biomasses ≤0, reasoning that models producing fewer negative values are more realistic.

Moreover, following Boit et al. (2012), we considered the similarity between our models and the empirical data by the Bray–Curtis dissimilarity (BC, Equations S5 and S6). In this, we considered the similarity between the recorded biomasses and the median (same as mean, in case of normal noise) of predicted biomasses by the model with environmental noise, summing over all guilds and years of data. In case of observation noise, we just compared the predicted deterministic dynamics to data. To consider the random variation in biomasses the model with environmental noise predicted, we also studied the coverage of the data by a central probability interval (CPI) of the predicted biomass distribution.

## 3 Results

### 3.1 Model Comparison and Similarity With Data

The Bray–Curtis dissimilarity indicated reasonable resemblance between the predictions about the average population dynamics and data. With environmental noise, we obtained 77% similarity between the median of the predicted biomasses and data (Table 1). With observation noise, the corresponding figure between the predicted deterministic dynamics and data was higher: 81%. The AIC value of the observation noise model was smaller than that of the environmental noise model (the difference was 24.48).

Although the Bray–Curtis similarity and AIC indicated a better fit for the observation noise model, the model with environmental noise produced a smaller number of predicted values that were ≤0 in terms of median biomasses (Table 1, Figures 1, 2). With environmental noise, we were able to obtain predictions with 2 years of median biomasses ≤0 (years 1998 and 2008 for eel) while with observation noise, the model, at its best, predicted extinction from an earlier year, 1996, onwards (for bream). The negative values remained in the predictions despite we set a penalty for biomasses ≤0 in model fitting. Despite the 2 years with median biomass ≤0, we regard the predictions by the environmental noise model reasonably accurate.

The best fitting environmental noise model was the model with absolute normal noise. This model obtained considerably lower AIC values and smaller numbers of predicted median biomasses ≤0 than the model with relative normal noise (Table 1 and Tables S4–S7). For observation noise, the difference between absolute and relative normal noise was not as high as for the environmental noise model (Table 1; Tables S10 and S11).

Although the average dynamics by the environmental noise model provided a reasonable fit to the data (Figures 1, 2), the model predicted considerably high probability of negative biomass values for almost all guilds. High probabilities of negative values also appeared for predictions about observable biomasses by the observation noise model. Nonetheless, the predicted variation resembled, overall, that of the data. When testing different penalizations of non‐positive biomass values in model fitting for environmental noise, other penalties than the one considering only median biomasses lead to poor model fit (Table S8). When testing lognormal noise by truncating the two remaining negative biomass values to be positive (Table S9), the fitted model predicted unrealistic fluctuations towards high biomasses (Figure S11). The Bray–Curtis dissimilarity indicated sligthly lower (74%) similarity between the data and predictions about the median dynamics for lognormal noise than for normal environmental noise.

For both environmental and observation noise, the best model was one without activity respiration (Tables S4, S5, S10 and S11) and when using the lower value of tested ones for the metabolic rates of zooplanktons and benthos (Tables S6 and S12). This was pronounced especially in terms of predicted biomass values ≤0. When comparing results with different feeding matrices, there were differences between the environmental and observation noise models. For environmental noise, the feeding matrix including cannibalism of perch and pike as well as white bream as a resoure for eel obtained the lowest AIC (Table S4), although there were no large differences in AIC values among the feeding matrices we tested (Table S4; the difference was ≤16.09). For observation noise, the feeding matrix without any of these feeding links yielded the best fit (Table S10, in terms of predicted biomass values ≤0). When testing different values of FR exponents, the differences in the AIC values were not very large for environmental noise (Table 1 and Table S7), but the model produced the lowest numbers of median biomass values ≤0 when we set the FR exponents to 0.3 or estimated them. The estimated values of FR exponents were often considerably high (Figure S10). For observation noise, differences in the model fit among different FR exponents were more considerable than for environmental noise (Table S13). For observation noise, we also obtained the best fit with the FR exponents set to 0.3, when considered in terms of both the AIC value and the number of predicted biomass values ≤0.

### 3.2 Parameter Estimates

There were differences in the estimated parameter values between the models tested (Figures 3 and 4; Figures S1–S9). For almost all guilds, however, the metabolic rates of consumers settled near their lower bounds (Figure S2). The exceptions were bream and smelt, for which the observation noise model yielded relatively high estimates of metabolic rates.

**FIGURE 3:**
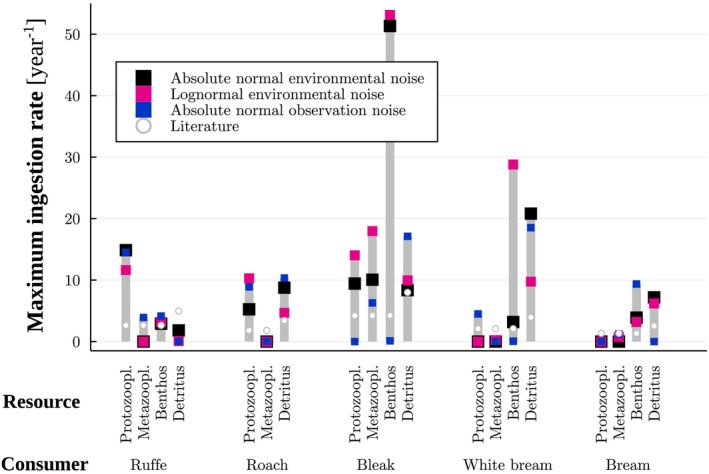
Maximum likelihood estimates of the half‐saturation constants (tonnes/km^2^) for different models. The estimates corresponds to results in Table 1 obtained with the functional response exponents set to 0.3 and using a penalty for or truncation of predicted biomass values ≤0.

**FIGURE 4:**
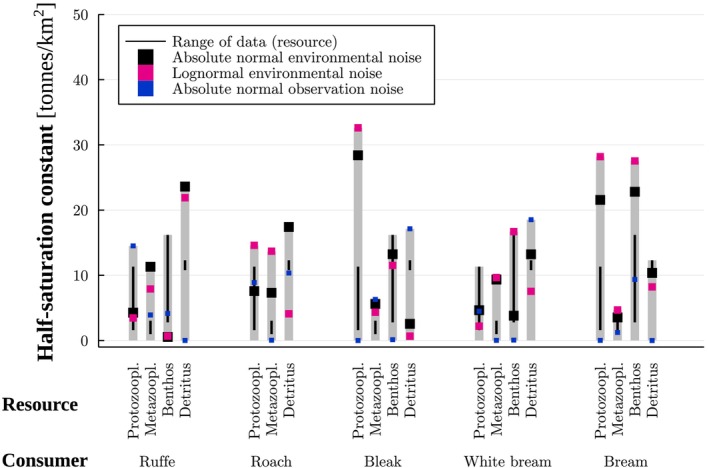
Maximum likelihood estimates of the maximum ingestion rates (year^−1^) for different models. The estimates correspond to the results in Table 1 obtained with the functional response exponents set to 0.3 and using a penalty for or truncation of predicted biomass values ≤0.

Among such cases where we obtained a guild‐specific maximum body weight (Table S1), the majority of the estimated maximum ingestion rates (Figure 3 and Figures S4–S6) agreed with previous studies. Indeed, 55%, 61%, and 52% of the obtained estimates, respectively, for normal and lognormal environmental noise and normal observation noise were higher than the values estimated following Williams et al. (2007), using the maximum body weights.

Regarding functional responses, the majority of estimated half‐saturation constants (Figure 4 and Figure S7–S9) were higher than the recorded biomass values of the resource guild (53%, 56% and 65%, respectively, for normal and lognormal environmental noise and normal observation noise). In some cases (13%–17% of the estimated values), the half‐saturation constants were lower than the data, and the rest (22%–33%) were within the range of recorded biomass values of the resource guild.

For all of the models, there was no considerable decrease in the estimated standard deviations compared to the standard deviation of the data (Figure S3). In some cases (e.g., benthos and detritus for normal environmental noise, ruffe and bream for observation noise) the estimated standard deviations were actually considerably higher than the sample ones. This corresponds to the obtained predictions, as for these guilds, the predicted underlying dynamics seemingly differed from the data (Figures 1 and 2).

## 4 Discussion

The present study used a trophic network model (Martinez 2020; Boit et al. 2012; Williams et al. 2007; Yodzis and Innes 1992) to describe the Lake Vortsjärv (Nõges and Nõges 2012) population dynamics. We calibrated this model to the Lake Võrtsjärv biomass time series (Bhele et al. 2022) utilizing numerical optimization methods and obtained 77%–81% similarity between the (average) modeled dynamics and recorded biomasses. This is similar to the figure (82%) Boit et al. (2012) obtained for the average growth season dynamics in Lake Constance. Despite the relatively high similarity we obtained, the model did not perform ideally in all cases as it predicted biomass values ≤0. This occurred for both types of noise we tested; environmental and observational. Of these two types of noise, we regard our model with environmental noise as a more promising starting point for future modeling of Lake Võrtsjärv dynamics than the observation noise model as it produced a smaller number of ≤0 biomass values in terms of average dynamics. These ≤0 biomass values occurred for eel, which is a special species in the Lake Võrtsjärv ecosystem in that the eel population is dependent on stocking (Cremona et al. 2018). Our model did not address stocking so that it may not be sufficient to describe the eel dynamics in the first place. Nonetheless, the fitting statistics (Table 1) between different noise types were in many cases contradictory, and our modeling approach must be regarded as tentative.

Although we regarded our model with environmental noise to capture the average dynamics of Võrtsjärv biomasses with reasonable accuracy, we had to conclude that the models we tested for noise—those assuming observation noise, as well as those incorporating environmental noise through stochastic differential equations—could not describe their annual variation realistically. For our best fitting environmental noise model, the one with absolute normal noise, the probability of a biomass value ≤0 was high for several annual predictions for almost all guilds. Nonetheless, the absolute noise model performed better than a relative noise model, although environmental noise is usually described to enter biological systems as Gaussian noise relative to abundances (Boettiger 2018). When testing a lognormal noise model, another traditional model for variation in abundances (Lande et al. 2003), we did not obtain sufficient similarity between the predicted average dynamics and the data, and the model predicted unrealistically large variation towards high biomasses.

The discrepancy between the modeled variation in abundances and recorded biomasses can stem from several reasons. First, we focused on the opposite extremes of noise assumptions (Hilborn and Mangel 1997)—only process noise or only observation noise—while the reality is something between. The inclusion of both process and observation noise would lead to a state‐space model (Newman et al. 2014). Although realistic as a model, separating in between observation noise and noise inherent in the process can be problematic in practice (Hilborn and Mangel 1997). Fitting a state‐space model may face a challenge with identification between process and observation noise (Auger‐Méthé et al. 2016). Such a problem might be alleviated by including information about one them to the model but we did not have prior information about what would be a realistic amount of observation or environmental noise for our model. Secondly, while we focused on testing perhaps the simplest formulation of environmental noise by independent Wiener processes, other choices for the noise process undoubtedly exist. For example, it is a question whether the Wiener processes describing guild‐specific noise processes should be independent or not, and the same holds for the increments of the noise process. Thirdly, we assumed stationary noise in the sense that the statistical properties of the noise process do not change in time but a more flexible model that allows the distribution of environmental noise to undergo temporal changes migth better capture the variation in abundances. Inclusion of both environmental and observation noise to the model as well as consideration of non‐stationarity or other noise processes than the Wiener process remain topics for future research.

Our study contributes to the calibration of bioenergetic models to empirical data using numerical optimization methods. Such a process has previously been applied to bioenergetic models with considerably fewer guilds and/or parameters (Banks et al. 2017a, 2017b); 15 guilds but only six unknown parameters—Koen‐Alonso and Yodzis (2005); four guilds and 38 estimated parameters. In model fitting, we kept most of the bioenergetic model parameters—the maximum growth rate of the producer, the metabolic and maximum ingestion rates of the consumers, and half‐saturation constants of the functional responses—as free parameters and estimated them, the variance parameters, and, in the case of the observation noise model, the initial biomasses by maximum likelihood estimation. While traditional bioenergetic models tie producer growth and consumer metabolic and ingestion rates to allometric rules, we employed allometric rules only to constrain the metabolic rates from below in fitting. Our approach originated from the uncertainty about the composition of guilds and consequently about the guild‐specific body masses. The end result was not completely satisfactory; the metabolic rates of consumers tended to settle near their lower boundaries and, although the freely estimated maximum ingestion rates mostly agreed with their corresponding lower boundaries, for a large part, they did not. As such, we must conclude that knowing better the guilds of Lake Võrtsjärv and what would be realistic ranges of their bioenergetic parameters could considerably benefit the modeling. The parameters of the functional responses are typically less well known (Boit et al. 2012). We also tested estimation of the functional response exponents, but this yielded unrealistically high values for them.

We acknowledge that allowing this degree of flexibility in parameter estimation may lead to overfitting, especially given the limited length of the time series. However, our primary goal was to demonstrate that a bioenergetic model can be closely aligned with empirical biomass dynamics. Testing the predictive power of the resulting models on independent data is beyond the scope of this study.

Regarding the parameter estimates we obtained, we notice that the model is high‐dimensional and complex, and the loss function can be multimodal (Tirronen and Kuparinen 2024). Nonetheless, the optimization method we used is well‐suited for these kinds of problems (Hansen 2016) and we carried out a large number of iterations with restarts in model fitting to further ensure a comprehensive search by the method. The range of the obtained estimates for all parameters except the metabolic rates reflects a decent coverage of the search space in optimization. Ideally, we would also have addressed the uncertainty of parameter estimates, but because of the complexity and dimensionality of the model, this was challenging using traditional procedures (Dalitz 2017) and remains a topic for future studies, possibly utilizing recently developed approaches for ATN models (Tirronen and Kuparinen 2024).

Validation of the bioenergetic model against empirical time series is limited to only a few studies (Banks et al. 2017a, 2017b; Boit et al. 2012; Koen‐Alonso and Yodzis 2005) and yet, only one of them considers such a model on the level of a whole food web (Boit et al. 2012). While Boit et al. (2012) calibrated their model to data manually, the present study explored possibilities to utilize numerical optimization methods in fitting a large trophic model to empirical data with a high number of parameters to be estimated. Our study shows that it is, in principle, possible to align such a model relatively closely with empirical biomass time series, but it also continues to demonstrate challenges that can be involved in inverse problems of bioenergetic models (Tirronen and Kuparinen 2024).

## Author Contributions

**Maria Tirronen:** conceptualization (equal), formal analysis (lead), methodology (lead), software (lead), visualization (lead), writing – original draft (lead), writing – review and editing (lead). **Anna Kuparinen:** conceptualization (equal), funding acquisition (lead).

## Conflicts of Interest

The authors declare no conflicts of interest.

## Supporting information


Data S1.

## Data Availability

The Võrtsjärv biomass time series have been published by Bhele et al. (2022). The computer codes for parameter estimation are available at https://zenodo.org/records/15619001.
