# Supplementary material for: Modeling Trophic Dynamics in Lake Võrtsjärv: Energy Flow and Species Interactions
Source: Ecol Evol. 2025 Jul 2;15(7):e71692. doi: 10.1002/ece3.71692 (PMC12222005; doi:10.1002/ece3.71692)
Supplement: Supplementary file 1 — Data S1. [file ECE3-15-e71692-s001.pdf]

Modeling trophic dynamics in Lake Võrtsjärv: Energy flow and species interactions

Supporting information

Maria Tirronen*[1,2] and Anna Kuparinen[1]

[1]*Department of Biological and Environmental Science, University of Jyväskylä, FI-40014 Jyväskylä, Finland*
[2]*Natural Resources Institute Finland (Luke), Survontie 9 A, FI-40500 Jyväskylä, Finland*

Contents

S1 Equations 2

S2 Lake Võrtsjärv food web 3

S3 Fixed and initial values of model parameters 4

S4 Model comparison and similarity with data 5

S5 Results for environmental noise 6
S5.1 Different feeding matrices without activity respiration 6
S5.2 Different feeding matrices with activity respiration 6
S5.3 $u^L_{m,2-4} = 10$ 7
S5.4 Different functional response exponents 7
S5.5 Penalty for predicted biomasses ≤ 0 8
S5.6 Lognormal noise 8

S6 Results for observation noise 9
S6.1 Different feeding matrices without activity respiration 9
S6.2 Different feeding matrices with activity respiration 9
S6.3 $u^L_{m,2-4} = 10$ 10
S6.4 Different functional response exponents 10

S7 Parameter estimates 11

S8 Predicted biomasses with lognormal environmental noise 21
. . .

*Corresponding author, email: maria.tirronen@gmail.com

S1 Equations

For the discretized model with absolute environmental noise, the transition density is

$$p\left[\overline{B}^*(t_k+1)\middle|\overline{B}^*(t_k),\overline{\theta}\right] = \prod_i N\left[B_i^*(t_k+1)\middle|B_i^*(t_k) + f_i\Big(\overline{B}^*(t_k),\overline{\theta}\Big)\Delta t; \sigma_i\sqrt{\Delta t}\right]. \tag{S1}$$

For absolute normal observation noise, the likelihood of the data reads as

$$\mathcal{L}\Big[\overline{B}^*(t_1),\ldots,\overline{B}^*(t_T)\Big|\overline{\theta}\Big] = \prod_{k=1}^{T}\prod_i N\left[B_i^*(t_k)\Big|B_i(t_k);\sigma_i\right]. \tag{S2}$$

For parameter estimation, we derived lower bounds for the metabolic rates (maintenance) of fish guilds utilizing allometry. Specifically, we derived the lower bounds using the species' maximum published weights (Froese and Pauly, 2023) and the formula (Williams et al., 2006)

$$u_{m,i}^L = a_{Ti}M_i^{-0.25}, \tag{S3}$$

where $a_{Ti} = 2.3$ [kg$^{0.25}$year^{-1}] and M_i stands for the adult body mass of the population. This yielded $u_{m,i}^L$ to range between 1-5 (Table S1).

S2 Lake Võrtsjärv food web

Table S1: The guilds in the Lake Võrtsjärv (Nõges and Nõges, 2012) food web, their feeding interactions following Cremona et al. (2018), maximum published weight (Froese and Pauly, 2023) and the lower bound set for the metabolic rate ($u^L_{m,i}$; Darwall et al., 2010; Williams et al., 2006). In our analysis, we tested the impact of the feeding links marked by * on model fitting.

ID	Guild	Description/scientific name	Diet	Max. weight [kg]	$u^L_{m,i}$ [day$^-1$]
1	Phytoplankton	Single-cell algae; filamentous algae; small, coccal algae; large, single-cell algae or colonies, diatoms	None	-	-
2	Protozooplankton	Ciliates	1-2, 15	-	1.0
3	Metazooplankton	Rotifers; Cladocerans; Cyclopoid copepods	1-2, 15	-	1.0
4	Benthos	Mostly chironomids	1, 15	-	1.0
5	Ruffe	*Gymnocephalus cernua*	2-4, 15	0.4	2.89
6	Roach	*Rutilus rutilus*	2-3, 15	1.8	1.99
7	Bleak	*Alburnus alburnus*	2-4, 15	0.06	4.65
8	White Bream	*Blicca bjoerkna*	2-4, 15	1.0	2.30
9	Bream	*Abramis brama*	2-4, 15	6.0	1.47
10	Smelt	*Osmerus eperlanus*	2-3, 15	0.178	3.54
11	Perch	*Perca fluviatilis*	2-10, 11*, 15	4.8	1.55
12	Eel	*Anguilla Anguilla*	2-7, 8*, 9-10, 15	6.6	1.43
13	Pikeperch	*Sander lucioperca*	2-11, 15	20.0	1.09
14	Pike	*Esox lucius*	2-3, 5-13, 14*, 15	28.4	1.00
15	Detritus	Dead particulate organic material	None	-	-

S3 Fixed and initial values of model parameters

Table S2: The fixed parameter values in the Lake Võrtsjärv trophic model (Table 1). The parameters are defined in Section 2.1 in the main document.

Parameter	Unit	Fixed value
s_i	-	0.2
$u_{a,i}$	-	0.4
e_{ij}	-	0.45 when j is phytoplankton or detritus<br>0.85 when j is zooplankton or fish

Table S3: Initial values for the parameters that we estimated for the Lake Võrtsjärv trophic model (Table 1). In model fitting, we parameterized the metabolic rates as $u^L_{m,i} + u_{m,i}$ (Table 1). For lognormal noise, we obtained initial σ_i based on log-transformed data. The parameters are defined in Sections 2.1–2.3 in the main document. $B^*_i(t)$ denotes the recorded biomass of the guild i at the year t.

Parameter	Unit	Initial values
r_i	year^{-1}	1.0
K	tonnes/km^2	$\max_t B_1(t)$
$u_{m,i}$	year^{-1}	1.0
J_{ij}	year^{-1}	1.0
q_{ij}	-	0.3
$B0_{ij}$	tonnes/km^2	mean$[B_j]$
$B_i(0)$	tonnes/km^2	$B^*_i(t_1)$
σ_i	tonnes/km^2	$0.5 \cdot \frac{\text{std}[B^*_i]}{\text{mean}[B^*_i]}$ for relative normal noise,<br>$0.5 \cdot \text{std}[B^*_i]$ for absolute normal noise

S4 Model comparison and similarity with data

We compared models using the Akaike information criterion (AIC), defined by

$$\mathrm{AIC} = 2k - 2\ln(\mathcal{L}^{\max}), \tag{S4}$$

where k is the number of estimated model parameters and $\mathcal{L}^{\max}$ is the maximized value of the likelihood function.

We also considered the similarity between models and the empirical data by the Bray-Curtis dissimilarity:

$$BC = 1 - 2\frac{\sum_i \sum_k \min\left[B_i^*(t_k+1), B_i^*(t_k) + f_i\left(\overline{B}^*(t_k)\right)\right]}{\sum_i \sum_k B_i^*(t_k+1) + B_i^*(t_k) + f_i\left(\overline{B}^*(t_k)\right)} \tag{S5}$$

for environmental noise and

$$BC = 1 - 2\frac{\sum_i \sum_k \min\left[B_i^*(t_k), B_i(t_k)\right]}{\sum_i \sum_k B_i^*(t_k) + B_i(t_k)} \tag{S6}$$

for observation noise.

S5 Results for environmental noise

S5.1 Different feeding matrices without activity respiration

Table S 4: Akaike information criterion (AIC) value, its difference to the minimum AIC (ΔAIC), the negative log-likelihood ($-\ln(\hat{L})$) and the number of predicted mean abundances ≤ 0 (excluding detritus and for all guilds, the first one in parenthesis) for the model with environmental noise, for different feeding matrices tested. In this, we tested the impact of the feeding links 12→8, 11→11 and 14→14 on the model dynamics, the feeding links otherwise set to those presented in Table 3 in the main document. The functional response exponents (q_{ij}) were set to 0.3.

Noise	Feeding matrix	AIC	ΔAIC	$-\ln(\hat{L})$	Predictions ≤ 0
Absolute normal	**12→8, 11→11 and 14→14 included**	1128.31	0	384.16	16 (15)
	Only 12→8 included	1141.72	13.41	394.86	29 (29)
	Only 11→11 and 14→14 included	1144.40	16.09	394.20	41 (41)
	None of the feeding links included	1135.81	7.50	393.90	32 (32)
Relative normal	12→8, 11→11 and 14→14 included	$8.56 \cdot 10^{15}$	$8.56 \cdot 10^{15}$	$4.28 \cdot 10^{15}$	127 (116)
	Only 12→8 included	$8.56 \cdot 10^{15}$	$8.56 \cdot 10^{15}$	$4.28 \cdot 10^{15}$	161 (152)
	None of the feeding links included	$8.56 \cdot 10^{15}$	$8.56 \cdot 10^{15}$	$4.28 \cdot 10^{15}$	166 (155)

S5.2 Different feeding matrices with activity respiration

Table S5: Akaike information criterion (AIC) value, its difference (ΔAIC) to **the minimum AIC in Table S4**, the negative log-likelihood ($-\ln(\hat{L})$) and the number of predicted mean abundances ≤ 0 (excluding detritus and for all guilds, the first one in parenthesis) for the model with environmental noise, when activity respiration was included in the model. In this, we tested the impact of the feeding links 12→8, 11→11 and 14→14 on the model dynamics, the feeding links otherwise set to those presented in Table 3 in the main document. The functional response exponents (q_{ij}) were set to 0.3.

Noise	Feeding matrix	AIC	ΔAIC	$-\ln(\hat{L})$	Predictions ≤ 0
Absolute normal	12→8, 11→11 and 14→14 included	1330.18	201.87	485.09	105 (105)
	Only 12→8 included	1310.11	181.80	479.06	103 (103)
	None of the feeding links included	1258.34	130.03	455.17	83 (83)
Relative normal	12→8, 11→11 and 14→14 included	$8.56 \cdot 10^{15}$	$8.56 \cdot 10^{15}$	$4.28 \cdot 10^{15}$	182 (170)
	Only 12→8 included	$8.56 \cdot 10^{15}$	$8.56 \cdot 10^{15}$	$4.28 \cdot 10^{15}$	169 (156)
	None of the feeding links included	$8.56 \cdot 10^{15}$	$8.56 \cdot 10^{15}$	$4.28 \cdot 10^{15}$	181 (170)

S5.3 $u^L_{m,2-4} = 10$

Table S6: Akaike information criterion (AIC) value, its difference (ΔAIC) to **the minimum AIC in Table S4**, the negative log-likelihood ($-\ln(\hat{L})$) and the number of predicted mean abundances ≤ 0 (excluding detritus and for all guilds, the first one in parenthesis) for the model with environmental noise, when setting the lower bound of metabolic rates ($u^L_{m,i}$) of guilds 2-4 to ten. The functional response exponents were set to 0.3 and the feeding matrix corresponds to the one that obtained the minimum AIC value with $u^L_{m,2-4} = 1$ (Table S4). No activity respiration was included in the model.

Noise	AIC	ΔAIC	$-\ln(\hat{L})$	Predictions ≤ 0
Absolute normal	1246.14	117.83	443.07	46 (46)
Relative normal	$8.56 \cdot 10^{15}$	$8.56 \cdot 10^{15}$	$4.28 \cdot 10^{15}$	172 (163)

S5.4 Different functional response exponents

Table S7: Akaike information criterion (AIC) value, its difference (ΔAIC) to **the minimum AIC in Table S4**, the negative log-likelihood ($-\ln(\hat{L})$), the number of predicted mean abundances ≤ 0 (excluding detritus and for all guilds, the first one in parenthesis) and the coefficient of variation (standard deviation divided by mean) of the predicted mean abundances for the model with environmental noise, for the functional response exponents (q_{ij}) tested. The feeding matrix corresponds to the one that obtained the minimum AIC value when functional response exponents were set to 0.3 with no activity respiration in the model and $u^L_{m,2-4} = 1$ (Table S4).

Noise	q_{ij}	AIC	ΔAIC	$-\ln(\hat{L})$	Predictions ≤ 0	CV
Absolute normal	Estimated	1140.27	11.96	315.13	17 (11)	1.39
	0	1162.75	34.44	401.37	32 (31)	1.27
	0.3	1128.31	0	384.16	16 (15)	1.29
	0.5	1140.38	12.07	390.19	24 (24)	1.26
	0.7	1108.68	-19.63	374.34	23 (22)	1.25
	1	1119.22	-9.09	379.61	23 (23)	1.32
Relative normal	Estimated	$3.73 \cdot 10^7$	$3.73 \cdot 10^7$	$1.87 \cdot 10^7$	190 (187)	-0.45
	0.3	$8.56 \cdot 10^{15}$	$8.56 \cdot 10^{15}$	$4.28 \cdot 10^{15}$	127 (116)	0.14

S5.5 Penalty for predicted biomasses ≤ 0

Table S8: Akaike information criterion (AIC) value, its difference (ΔAIC) to **the minimum AIC in Table S4**, the negative log-likelihood ($-\ln(\hat{L})$) and the number of predicted mean abundances ≤ 0 (excluding detritus and for all guilds, the first one in parenthesis) for the model with absolute normal environmental noise, when the loss function was set to include a penalty for biomass values ≤ 0 in model fitting. The functional response exponents (q_{ij}) were set to 0.3 and the feeding matrix corresponds to the one that obtained the minimum AIC value with no activity respiration in the model and $u^L_{m,2-4} = 1$ (Table S4).

Penalty criterion	AIC	ΔAIC	$-\ln(\hat{L})$	Predictions ≤ 0
Mean biomass ≤ 0	1171.25	42.94	405.62	2 (2)
0.3th percentile of biomass ≤ 0	4636.59	3508.28	2138.30	6 (6)
0.1th percentile of biomass ≤ 0	5529.67	4401.36	2584.84	5 (5)
0.01th percentile of biomass ≤ 0	6142.53	5014.22	2891.26	7 (5)

S5.6 Lognormal noise

Table S9: Akaike information criterion (AIC) value, its difference (ΔAIC) to **the minimum AIC in Table S4** and the negative log-likelihood ($-\ln(\hat{L})$) for lognormal environmental noise. The feeding matrix corresponds to the one that obtained the minimum AIC value for absolute normal environmental noise when functional response exponents were set to 0.3 with no activity respiration in the model and $u^L_{m,2-4} = 1$ (Table S4). The initial values of the parameters were set to those obtained when penalty of non-positivity was set for mean biomasses for the model with absolute normal environmental noise (Table S8). The year 1998 and 2008 biomasses of eel were truncated from ≤ 0 to 10^{-8}.

Noise	q_{ij}	AIC	ΔAIC	$-\ln(\hat{L})$	Median predictions ≤ 0 (truncated to 10^{-8})
Absolute lognormal	0.3	1057.38	-70.93	348.69	2 (2)
Relative lognormal	0.3	12556.56	11428.25	6098.28	2 (2)

S6 Results for observation noise

S6.1 Different feeding matrices without activity respiration

Table S10: Akaike information criterion (AIC) value, its difference (ΔAIC) to **the minimum AIC in Table S4**, the negative log-likelihood ($-\ln(\hat{L})$) and the number of predicted abundances ≤ 0 (excluding detritus and for all guilds, the first one in parenthesis) for the model with observation noise, for different feeding matrices tested. In this, we tested the impact of the feeding links 12→8, 11→11 and 14→14 on the model dynamics, the feeding links otherwise set to those presented in Table 3 in the main document. The functional response exponents (q_{ij}) were set to 0.3.

Noise	Feeding matrix	AIC	ΔAIC	$-\ln(\hat{L})$	Predictions ≤ 0
Absolute normal	12→8, 11→11 and 14→14 included	1218.66	90.35	414.33	68 (68)
	Only 12→8 included	1160.93	32.62	389.46	51 (51)
	Only 11→11 and 14→14 included	1192.28	63.92	403.14	50 (50)
	**None of the feeding links included**	1181.16	52.85	401.58	17 (17)
Relative normal	12→8, 11→11 and 14→14 included	931.40	-196.91	270.70	31 (31)
	None of the feeding links included	872.66	-255.65	247.33	83 (83)

S6.2 Different feeding matrices with activity respiration

Table S11: Akaike information criterion (AIC) value, its difference (ΔAIC) to **the minimum AIC in Table S4**, the negative log-likelihood ($-\ln(\hat{L})$) and the number of predicted abundances ≤ 0 (excluding detritus and for all guilds, the first one in parenthesis) for different feeding matrices for the model with observation noise, the trophic model including activity respiration. The functional response exponents were set to 0.3.

Noise	Feeding matrix	AIC	ΔAIC	$-\ln(\hat{L})$	Predictions ≤ 0
Absolute normal	12→8, 11→11 and 14→14 included	1290.12	161.81	450.06	118 (118)
	None of the feeding links included	1313.63	185.32	467.81	135 (135)
Relative normal	12→8, 11→11 and 14→14 included	910.28	-218.03	260.14	168 (168)
	None of the feeding links included	922.42	-205.89	272.21	169 (169)

S6.3 $u^L_{m,2-4} = 10$

Table S12: Akaike information criterion (AIC) value, its difference (ΔAIC) to **the minimum AIC in Table S4**, the negative log-likelihood ($-\ln(\hat{L})$) and the number of predicted abundances ≤ 0 (excluding detritus and for all guilds, the first one in parenthesis) for the model with observation noise, when setting the lower bound of metabolic rates ($u^L_{m,i}$) of guilds 2-4 to ten. The functional response exponents were set to 0.3 and the feeding matrix corresponds to the one that obtained the minimum AIC value with $u^L_{m,2-4} = 1$ (Table S10). No activity respiration was included in the model.

Noise	AIC	ΔAIC	$-\ln(\hat{L})$	Predictions ≤ 0
Absolute normal	1727.30	598.99	674.65	117 (117)

S6.4 Different functional response exponents

Table S13: Akaike information criterion (AIC) value, its difference (ΔAIC) to **the minimum AIC in Table S4**, the negative log-likelihood ($-\ln(\hat{L})$), the number of predicted abundances ≤ 0 (excluding detritus and for all guilds, the first one in parenthesis) and the coefficient of variation (standard deviation divided by mean) of the predicted abundances for the model with observation noise, for the functional response exponents (q_{ij}) tested. The feeding matrix corresponds to the one that obtained the minimum AIC value when functional response exponents were set to 0.3 with no activity respiration in the model and $u^L_{m,2-4} = 1$ (Table S10). For $q = 0.3$, iteration was increased from 1000 to 3000 (marked by *).

Noise	q_{ij}	AIC	ΔAIC	$-\ln(\hat{L})$	Predictions ≤ 0	CV
Absolute normal	Estimated	-30361.12	-31489.43	15441.56	226 (225)	0.33
	0	1211.60	83.29	416.80	68 (68)	5.65
	0.3*	1146.77	18.46	384.39	17 (17)	5.01
	0.5	1148.82	20.51	385.41	51 (51)	4.20
	0.7	1094.75	-33.56	358.38	34 (34)	4.68
	1	1297.49	169.18	459.74	17 (17)	5.61

S7 Parameter estimates

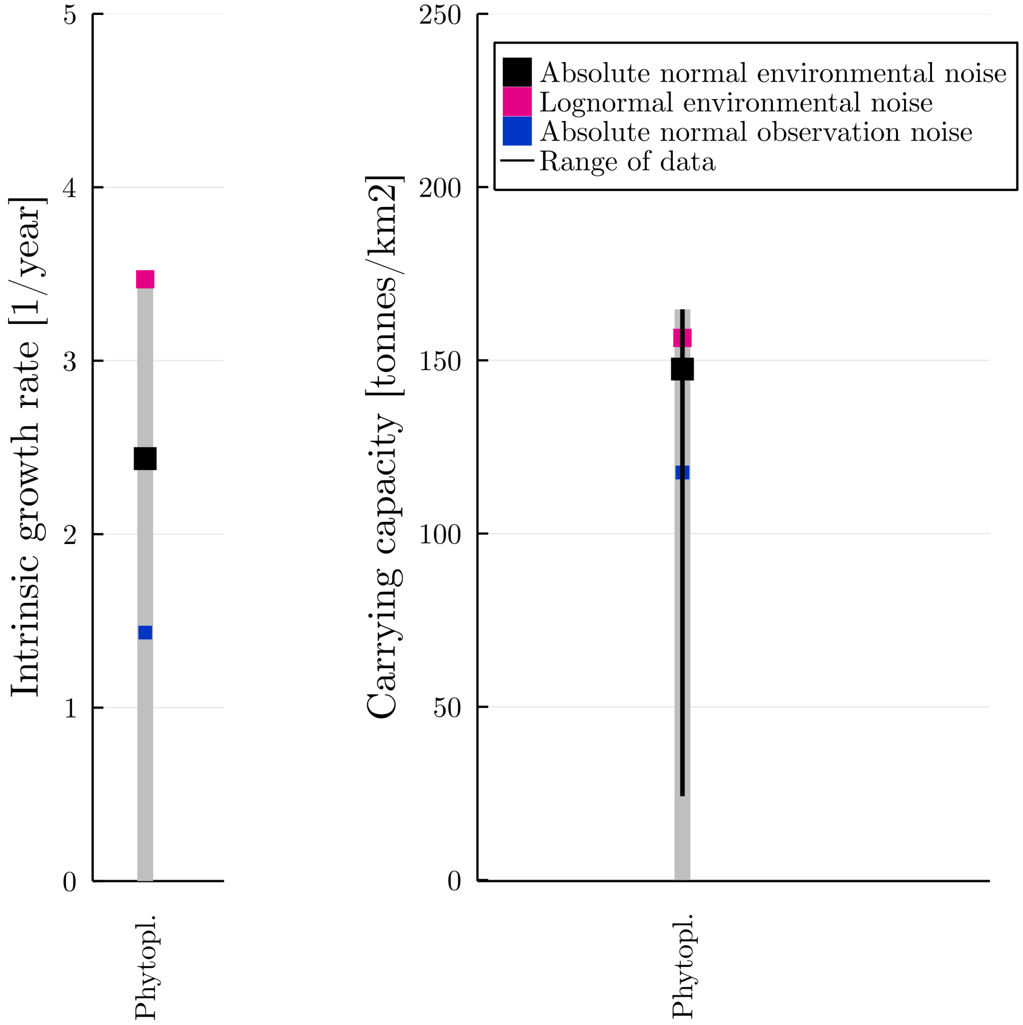

Figure S1: Maximum likelihood estimates of the intrinsic growth rate (year^{-1}) of the producer guild (phytoplankton) and of the carrying capacity (tonnes/km^2) for the producer for different models. The estimates correspond to the results in Table 1 of the main document, obtained with the functional response exponents set to 0.3 and using a penalty for or truncation of predictions ≤ 0 about the dynamics.

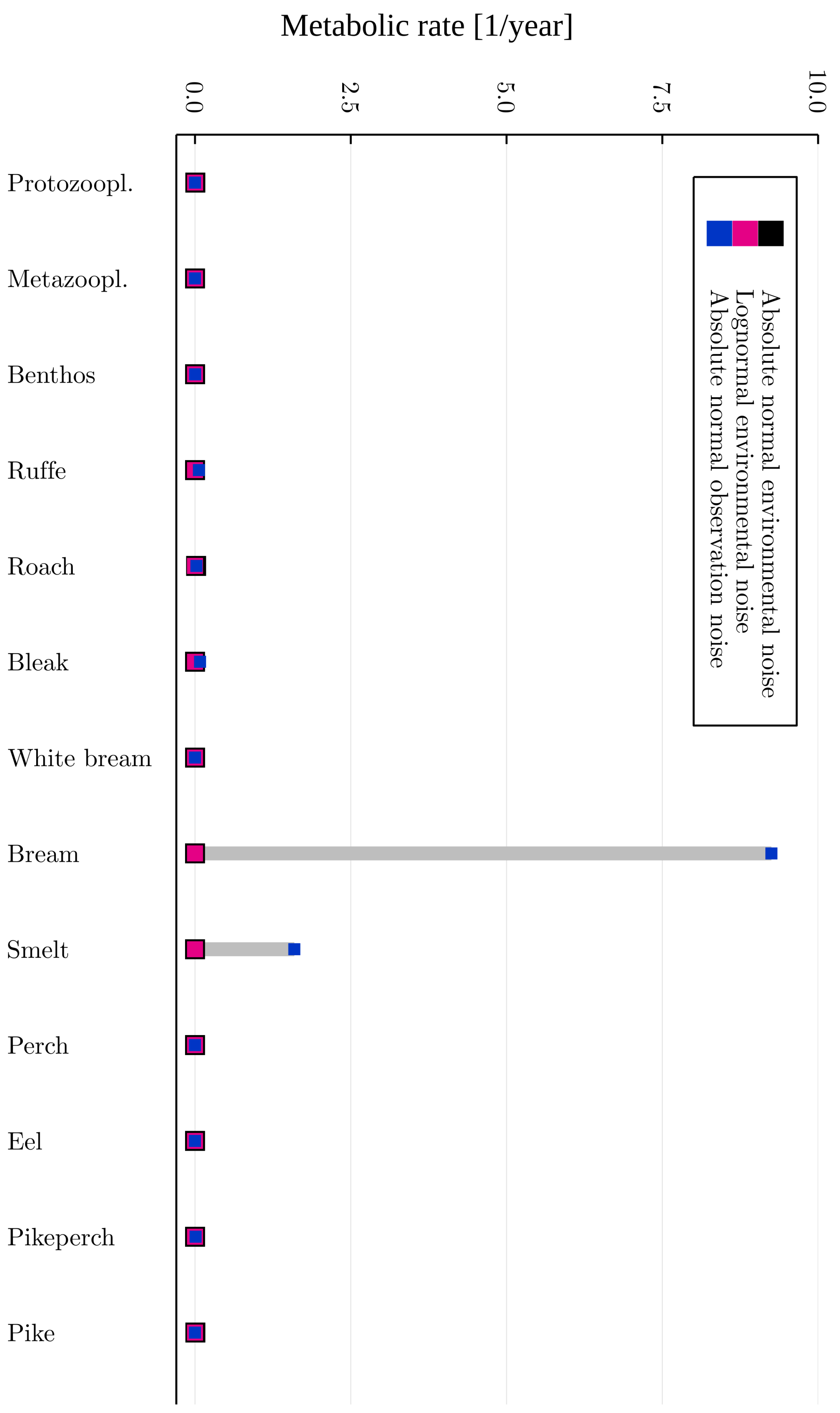

Figure S2: Maximum likelihood estimates of the metabolic rates (year^{-1}) of the consumer guilds for different models. The estimates correspond to the results in Table 1 of the main document, obtained with the functional response exponents set to 0.3 and using a penalty for or truncation of predictions ≤ 0 about the dynamics.

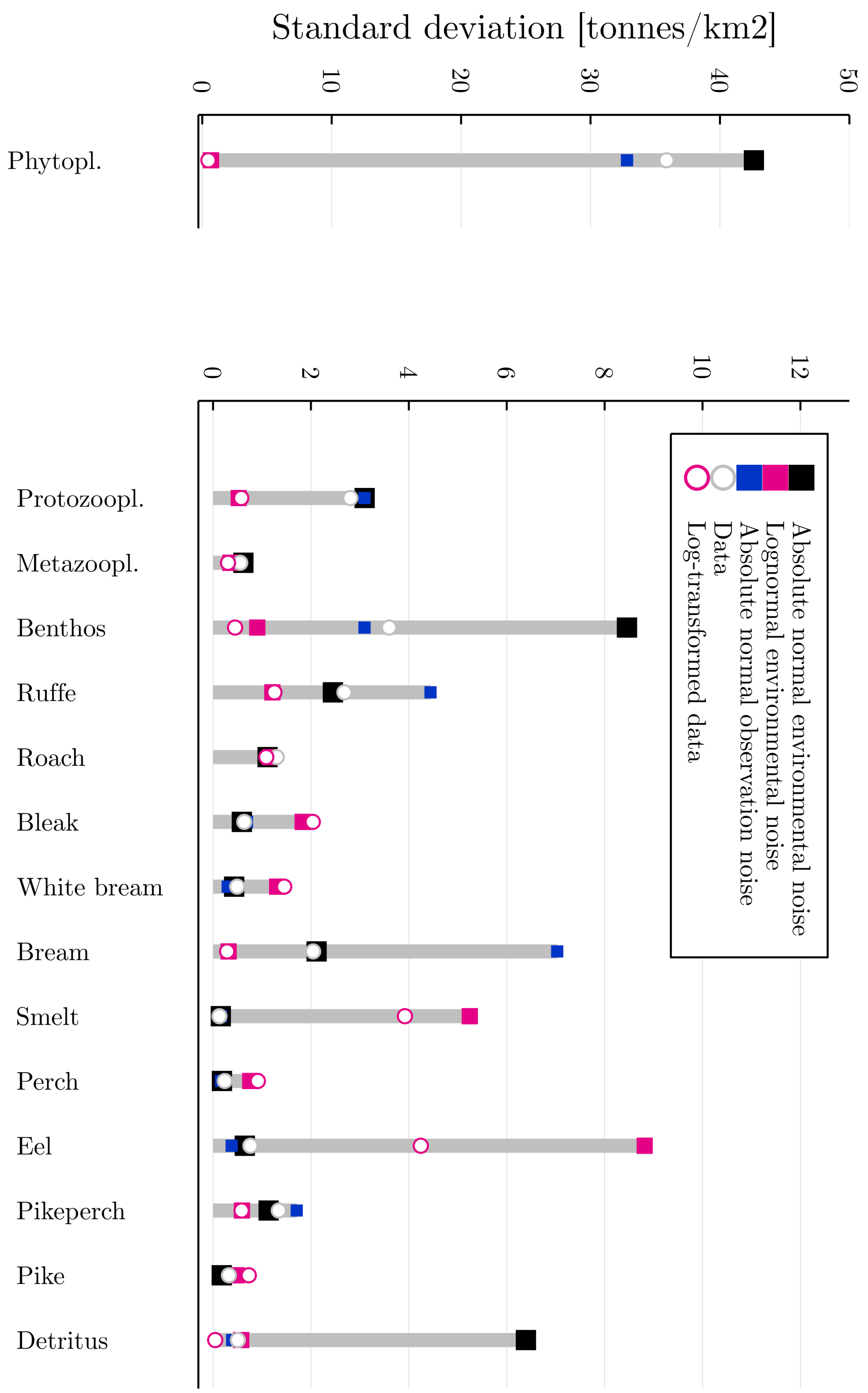

Figure S3: Maximum likelihood estimates of the standard deviations (tonnes/km^2) for different models. The estimates correspond to the results in Table 1 of the main document, obtained with the functional response exponents set to 0.3 and using a penalty for or truncation of predictions ≤ 0 about the dynamics.

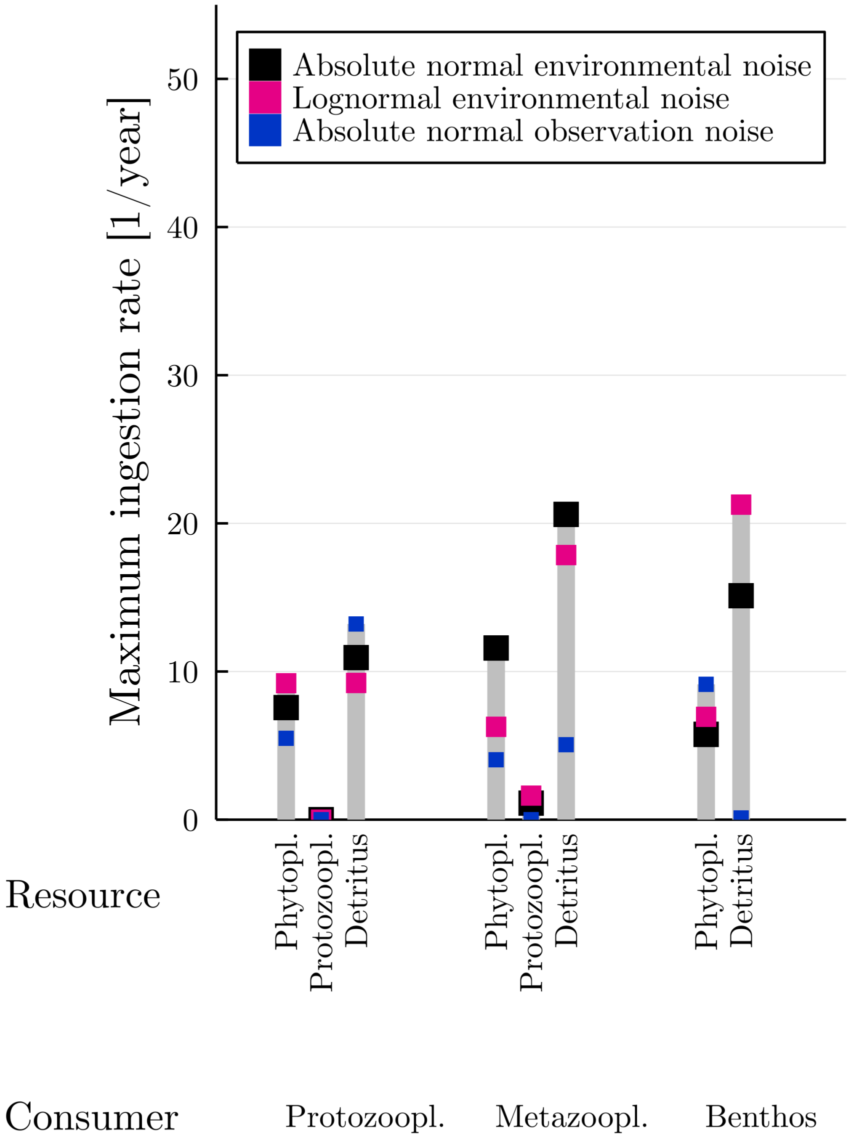

Figure S4: Maximum likelihood estimates of the maximum ingestion rates (year^{-1}) for different models. The estimates correspond to the results in Table 1 of the main document, obtained with the functional response exponents set to 0.3 and using a penalty for or truncation of predictions ≤ 0 about the dynamics.

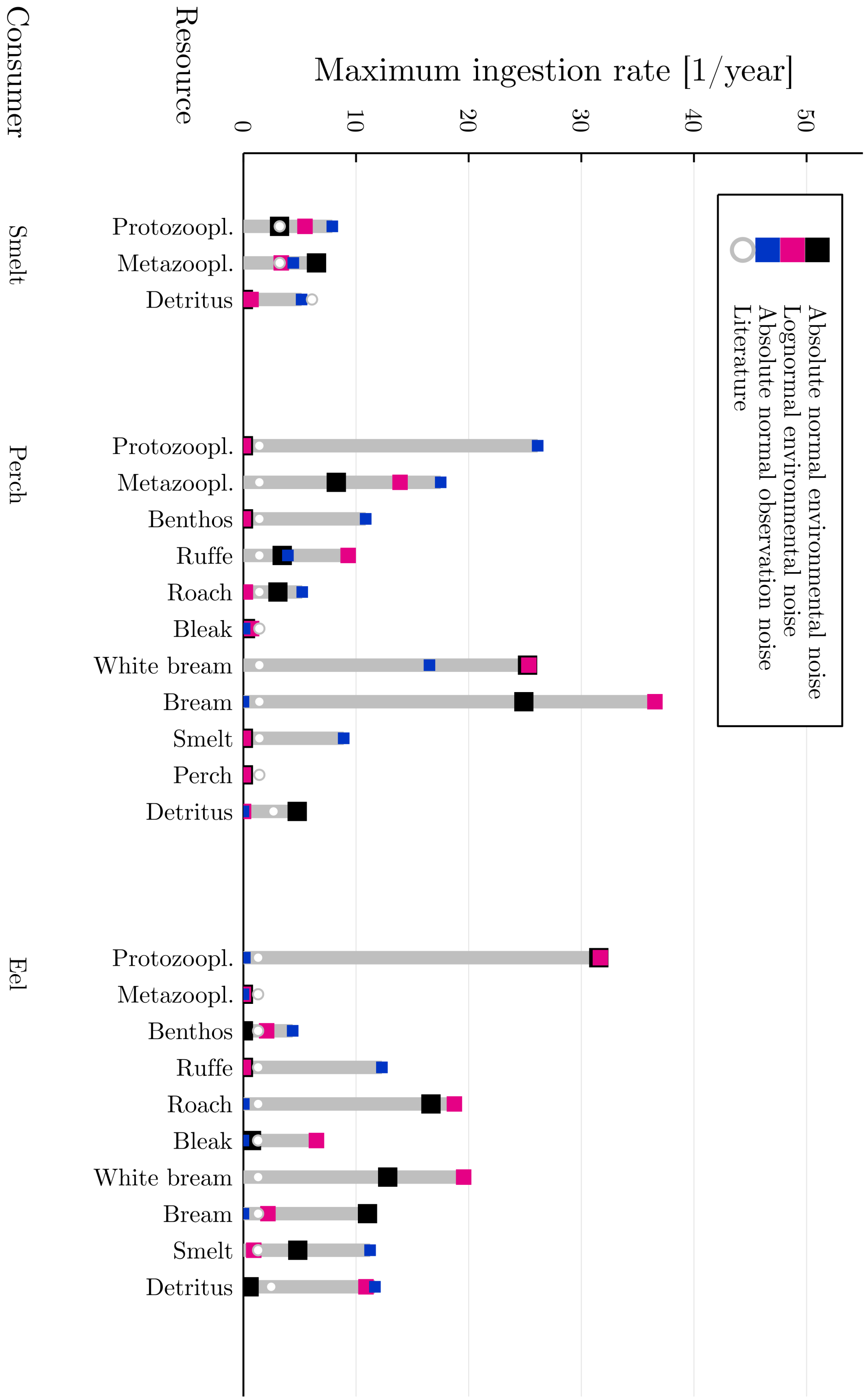

Figure S5: Maximum likelihood estimates of the maximum ingestion rates (year^{-1}) for different models. The estimates correspond to the results in Table 1 of the main document, obtained with the functional response exponents set to 0.3 and using a penalty for or truncation of predictions ≤ 0 about the dynamics.

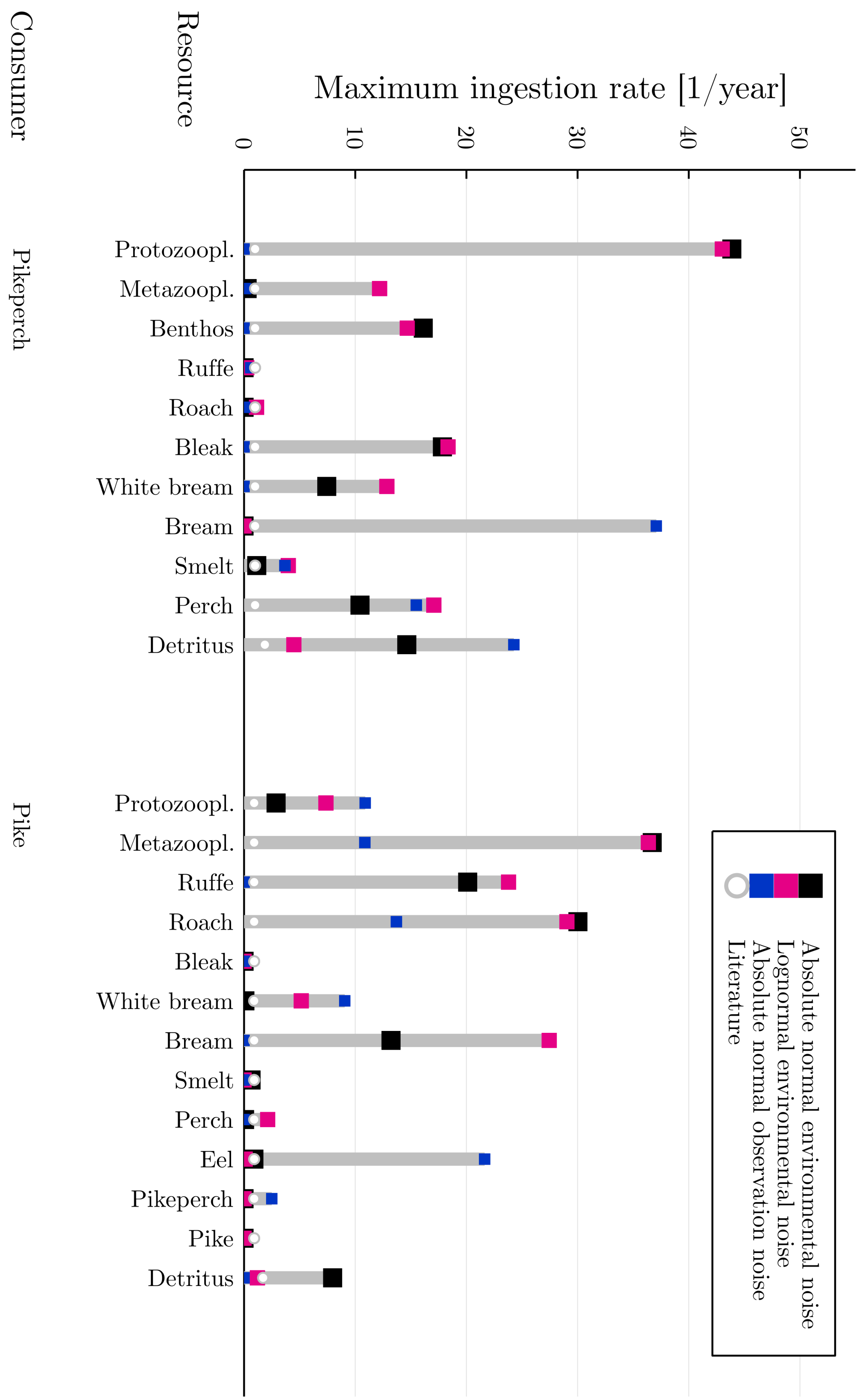

Figure S6: Maximum likelihood estimates of the maximum ingestion rates (year^{-1}) for different models. The estimates correspond to the results in Table 1 of the main document, obtained with the functional response exponents set to 0.3 and using a penalty for or truncation of predictions ≤ 0 about the dynamics.

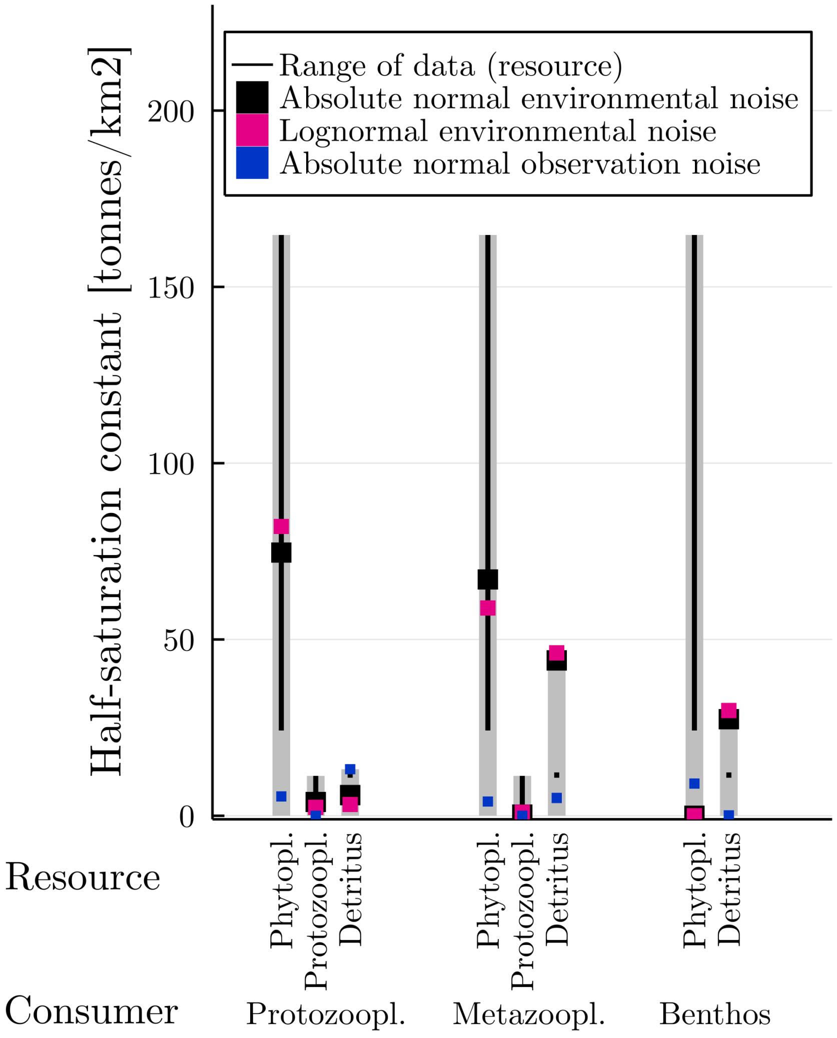

Figure S7: Maximum likelihood estimates of the half-saturation constants (tonnes/km^2) for different models. The estimates corresponds to results in Table 4 obtained with the functional response exponents set to 0.3 and using a penalty for or truncation of predictions ≤ 0 about the dynamics.

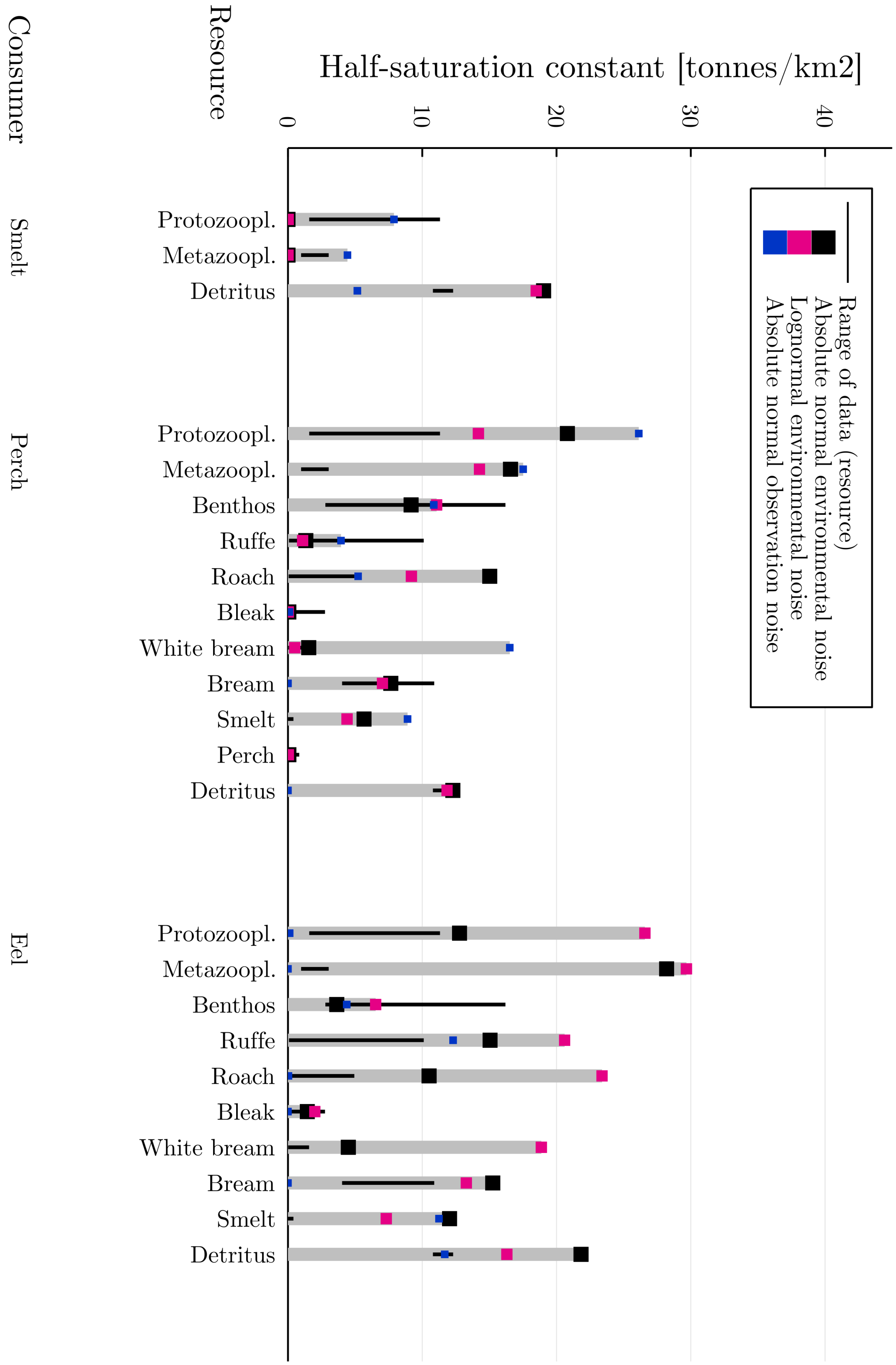

Figure S8: Maximum likelihood estimates of the half-saturation constants (tonnes/km^2) for different models. The estimates corresponds to results in Table 4 obtained with the functional response exponents set to 0.3 and using a penalty for or truncation of predictions ≤ 0 about the dynamics.

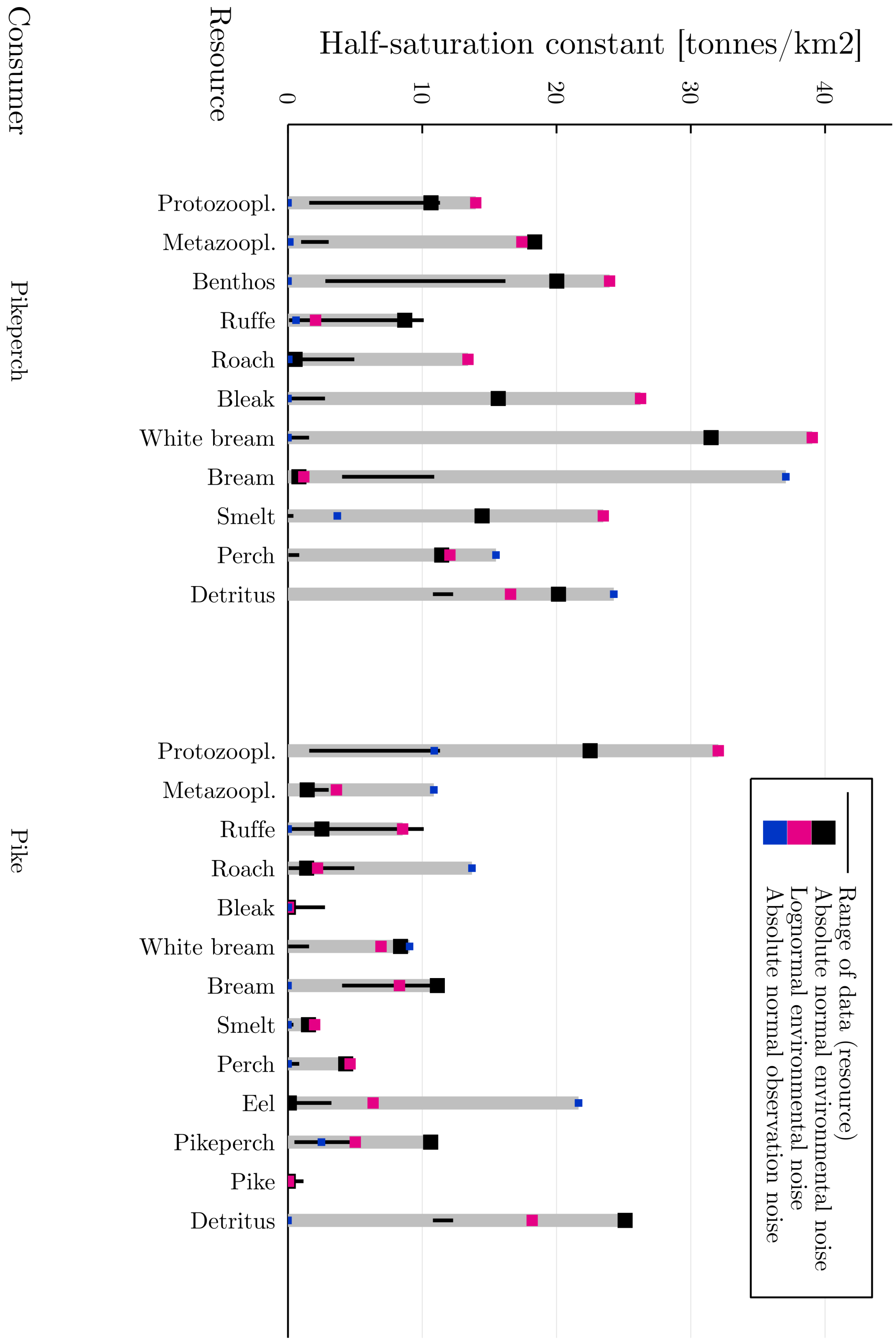

Figure S9: Maximum likelihood estimates of the half-saturation constants (tonnes/km^2) for different models. The estimates corresponds to results in Table 4 obtained with the functional response exponents set to 0.3 and using a penalty for or truncation of predictions ≤ 0 about the dynamics.

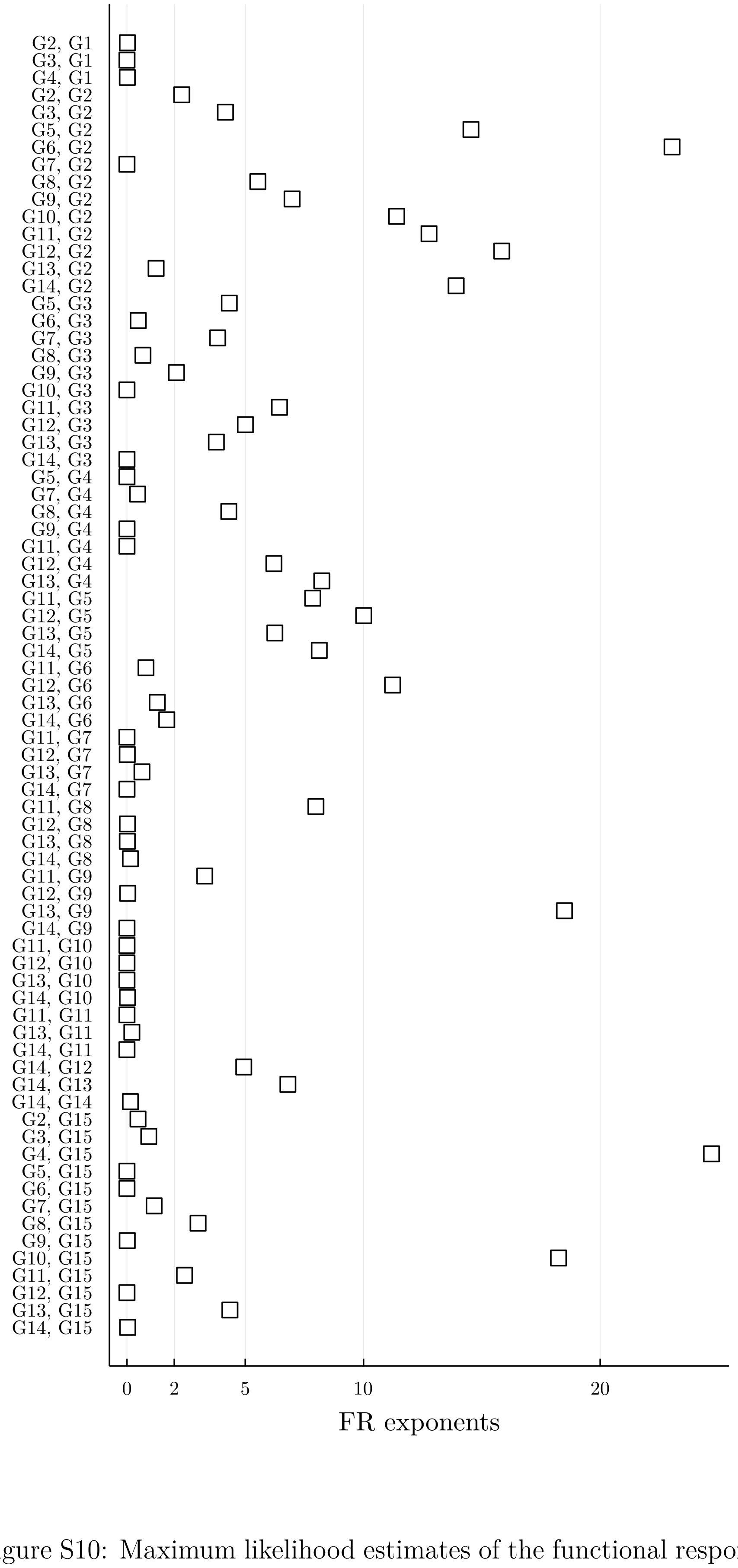

Figure S10: Maximum likelihood estimates of the functional response exponents (the consumer guild is given first, the resource guild is given after comma). The figure presents the results for the model with absolute normal environmental noise. The feeding matrix includes cannibalism of perch and pike and white bream as a resoure for eel. The model includes no activity respiration and the lower bounds of the metabolic rates of zooplanktons and benthos were set to one.

S8 Predicted biomasses with lognormal environmental noise

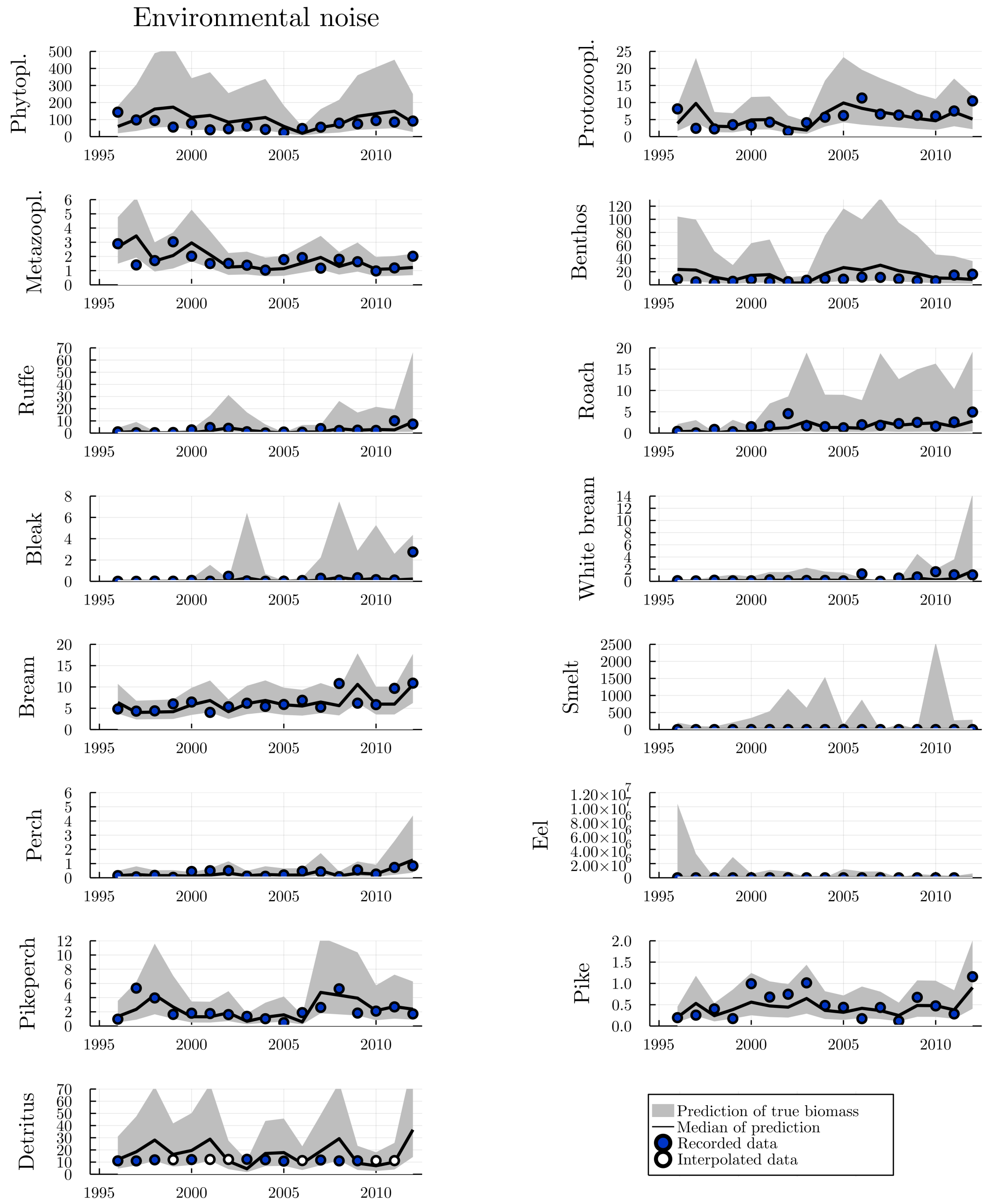

Figure S11: Recorded and interpolated biomass (tonnes/km^2) data used in model fitting, and the predicted biomasses for different guilds by the trophic model with absolute log-normal environmental noise. The feeding matrix includes cannibalism of perch and pike and white bream as a resoure for eel. The model includes no activity respiration, the lower bounds of the metabolic rates of zooplanktons and benthos were set to one and the functional response exponents were set to 0.3. The year 1998 and 2008 biomasses of eel were truncated from ≤ 0 to 10^8. The filled areas correspond to the 90 % central probability intervals of the predicted biomasses.

References

F. Cremona, A. Järvalt, U. Bhele, H. Timm, S. Seller, J. Haberman, P. Zingel, H. Agasild, P. Nõges, and T. Nõges. Relationships between fisheries, foodweb structure, and detrital pathway in a large shallow lake. *Hydrobiologia*, 820:145–163, 09 2018. doi: 10.1007/s10750-018-3648-2.

W. Darwall, E. Allison, G. Turner, and K. Irvine. Lake of flies, or lake of fish? A trophic model of Lake Malawi. *Ecological Modelling*, 221:713–727, 02 2010. doi: 10.1016/j.ecolmodel.2009.11.001.

R. Froese and D. Pauly. FishBase. World Wide Web electronic publication, 2023. URL `www.fishbase.org`. Version (10/2023).

P. Nõges and T. Nõges. *Võrtsjärv lake in Estonia*, pages 850–861. 01 2012. doi: 10.1007/978-1-4020-4410-6_228.

R. Williams, U. Brose, and N. Martinez. *Homage to Yodzis and Innes 1992: Scaling up Feeding-Based Population Dynamics to Complex Ecological Networks*, pages 37–51. Springer Netherlands, 01 2006. ISBN 978-1-4020-5336-8. doi: 10.1007/978-1-4020-5337-5_2.
